# The Histone H3K36 Methyltransferase MES-4 Acts Epigenetically to Transmit the Memory of Germline Gene Expression to Progeny

**DOI:** 10.1371/journal.pgen.1001091

**Published:** 2010-09-02

**Authors:** Andreas Rechtsteiner, Sevinc Ercan, Teruaki Takasaki, Taryn M. Phippen, Thea A. Egelhofer, Wenchao Wang, Hiroshi Kimura, Jason D. Lieb, Susan Strome

**Affiliations:** 1 Department of Molecular, Cell, and Developmental Biology, University of California Santa Cruz, Santa Cruz, California, United States of America; 2 Department of Biology, Carolina Center for Genome Sciences and Lineberger Comprehensive Cancer Center, University of North Carolina at Chapel Hill, Chapel Hill, North Carolina, United States of America; 3 Graduate School for Frontier Biosciences, Osaka University, Suita, Osaka, Japan; The Babraham Institute, United Kingdom

## Abstract

Methylation of histone H3K36 in higher eukaryotes is mediated by multiple methyltransferases. Set2-related H3K36 methyltransferases are targeted to genes by association with RNA Polymerase II and are involved in preventing aberrant transcription initiation within the body of genes. The targeting and roles of the NSD family of mammalian H3K36 methyltransferases, known to be involved in human developmental disorders and oncogenesis, are not known. We used genome-wide chromatin immunoprecipitation (ChIP) to investigate the targeting and roles of the *Caenorhabditis elegans* NSD homolog MES-4, which is maternally provided to progeny and is required for the survival of nascent germ cells. ChIP analysis in early *C. elegans* embryos revealed that, consistent with immunostaining results, MES-4 binding sites are concentrated on the autosomes and the leftmost ∼2% (300 kb) of the X chromosome. MES-4 overlies the coding regions of approximately 5,000 genes, with a modest elevation in the 5′ regions of gene bodies. Although MES-4 is generally found over Pol II-bound genes, analysis of gene sets with different temporal-spatial patterns of expression revealed that Pol II association with genes is neither necessary nor sufficient to recruit MES-4. In early embryos, MES-4 associates with genes that were previously expressed in the maternal germ line, an interaction that does not require continued association of Pol II with those loci. Conversely, Pol II association with genes newly expressed in embryos does not lead to recruitment of MES-4 to those genes. These and other findings suggest that MES-4, and perhaps the related mammalian NSD proteins, provide an epigenetic function for H3K36 methylation that is novel and likely to be unrelated to ongoing transcription. We propose that MES-4 transmits the memory of gene expression in the parental germ line to offspring and that this memory role is critical for the PGCs to execute a proper germline program.

## Author Summary

Germ cells transmit the genome from one generation to the next. The identity and immortality of germ cells are crucial for the perpetuation of species, yet the mechanisms that regulate these properties remain elusive. In *C.elegans*, a histone methyltransferase MES-4 is required for survival of the primordial germ cells. MES-4 methylates histone H3 at lysine 36 (H3K36), a modification previously linked to transcription elongation and involved in preventing aberrant transcription initiation within the body of genes. Surprisingly, our genome-wide analysis of MES-4 binding sites in *C. elegans* embryos revealed that MES-4 is capable of associating with genes that were expressed in the germ line of the parent worms but are no longer being actively transcribed in embryos. To our knowledge, this is the first example of transcription-uncoupled H3K36 methylation. We suggest that MES-4-generated H3K36 methylation serves an “epigenetic role,” by marking germline-expressed genes and by carrying the memory of gene expression from one generation of germ cells to the next.

## Introduction

Regulation of gene expression through DNA packaging into chromatin has emerged as an important theme in development (reviewed in [1]). Chromatin consists of nucleosomal units of 147 bp segments of DNA wrapped around histone octamers composed of H2A, H2B, H3, and H4 [1]–[3]. Post-translational modification of histones via methylation, acetylation and addition of other covalent marks constitutes an important mechanism to regulate chromatin structure, DNA accessibility, and recruitment of regulatory factors during transcription [4], [5]. Some modifications, such as H3K27 methylation, additionally serve epigenetic roles by propagating particular chromatin states and gene expression patterns from mother to daughter cells [e.g. 6], [7].

This paper focuses on MES-4, a *C. elegans* enzyme that methylates histone H3 on lysine 36 (H3K36me) [8]. In yeast, all H3K36 methylation is carried out by the SET domain-containing protein Set2 [9]. In more complex eukaryotes, H3K36 methylation is carried out by two groups of enzymes. The first group includes the Set2-related proteins, called MET-1 in *C. elegans*, Hypb/Set2 in fruit flies, and HYPB/Setd2 in mammals, [10]–[13]. The second group includes MES-4-related proteins called MES-4 in *C. elegans*, dMES-4 in fruit flies, and NSD1 (for nuclear receptor-binding SET domain protein 1), NSD2/WHSC1/MMSET, and NSD3/WHSC1L1 in mammals [11], [14]–[16]. Mutations in the human MES-4/NSD genes underlie developmental disorders and various human malignancies [15]–[17].

In *C. elegans*, the MES-4 histone methyltransferase (HMT) is essential for germ cell viability. The genetics of *mes-4* demonstrate that MES-4 serves a transgenerational (mother to offspring) role. Maternal *mes-4*(+) enables even homozygous *mes-4/mes-4* progeny to form a fully functional germ line, while absence of maternal *mes-4*(+) severely impairs the ability of the primordial germ cells (PGCs) in progeny to proliferate and causes them to deteriorate early [18]. Based on immunostaining of germ lines and embryos, MES-4 and the H3K36me2 marks generated by MES-4 are dramatically concentrated on the autosomes and absent from all but the left tip of the X chromosomes [8], [19]. Surprisingly, microarray analysis of adult germ lines from fertile *mes-4/mes-4* mothers revealed up-regulation of numerous (345) X-linked genes, along with mis-regulation of autosomal genes (155 up- and 115 down-regulated) (data from [8]; our unpublished results) suggesting that MES-4 participates in silencing the X chromosomes in wild-type adult germ lines [8], [20], [21]. Learning how MES-4 and H3K36 methylation promote PGC survival hinges on identifying the targets of MES-4 binding at the gene level and elucidating the role of MES-4-generated H3K36 methyl marks.

Studies of yeast Set2 have established one paradigm for binding and function of H3K36 HMTs. Yeast Set2 associates via its “SRI domain” with the C-terminal domain of RNA Polymerase II (Pol II) during the elongation phase of transcription and methylates H3K36 within the body of genes, predominantly in the 3′ coding region [22]–[26]. Set2-catalyzed H3K36me marks are recognized by the chromodomain of Eaf3 and the plant homeo domain (PHD) of Rco1, both in a complex with the Rpd3 enzyme that deacetylates histones. Delivery of Rpd3 to the body of genes suppresses spurious transcription initiation within the coding region [27]–[29]. Studies of X-chromosome dosage compensation in *Drosophila* have revealed another role for H3K36 methylation in mediating spreading of the dosage compensation complex from sites of initial recruitment [12], [30].

To investigate the targeting and roles of MES-4 in *C. elegans*, we employed ChIP-chip to determine the genome-wide distribution at high resolution of MES-4, H3K36me2, H3K36me3, and Pol II in extracts from early-stage embryos. Our analysis revealed that MES-4 accumulates over the bodies of ∼20% of genes, and that the majority of MES-4-bound genes are on the five autosomes and the left tip of the X. Comparing MES-4 and Pol II association with classes of genes with different temporal-spatial patterns of expression revealed that MES-4 resides on genes expressed in the maternal germ line and not genes newly expressed in early embryos. These findings, especially the observation that genes specifically expressed in the adult germ line are bound by MES-4 but not Pol II in early embryos, point to a mode of MES-4 recruitment that is different than Set2. MES-4 associates with and maintains methylation of germline-expressed genes through cell division and in a transcription-independent manner, establishing a novel epigenetic role for H3K36 methylation.

## Results

### MES-4, but not MET-1, is required for early-embryo maintenance of H3K36 trimethylation in the absence of RNA Polymerase II

**Figure 1:**
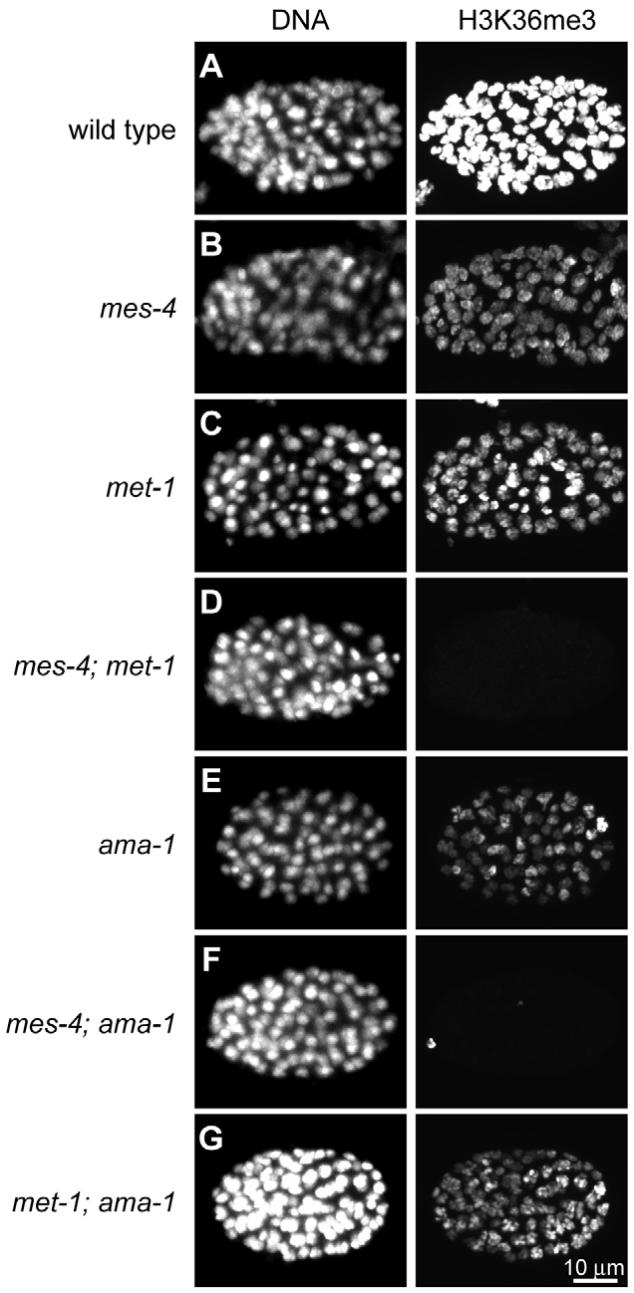
Two *C. elegans* HMTs, MES-4 and MET-1, perform H3K36 trimethylation and display different dependence on Pol II. H3K36me3 levels are reduced relative to wild type (A) in *mes-4* mutant (B), *met-1* mutant (C), and *ama-1(RNAi)* (E) ∼80–110-cell embryos. H3K36me3 is undetectable in *mes-4*; *met-1* (D) and *mes-4*; *ama-1(RNAi)* (F), but is still present in *met-1*; *ama-1(RNAi)* (G) embryos. Embryos were stained in parallel and images acquired and processed identically. See Figure S1 for specificity of H3K36me3 antibody.

Previous studies demonstrated that MES-4 generates H3K36me2 in adult germ lines and embryos [8], and the Set2 ortholog MET-1 generates H3K36me3 in embryos [10]. Our immunostaining experiments demonstrate that MES-4 contributes significantly to H3K36me3 as well, since H3K36me3 signal is abolished only in double mutant germ lines and embryos lacking both MES-4 and MET-1 (Figure 1A–1D; germline data not shown). To investigate the roles served by these two H3K36me3 HMTs, we tested the dependence of each HMT on RNA Pol II.

Previous studies suggested a Pol II-independent component of MES-4 function. MES-4-generated H3K36me2 immunofluorescence signal in embryos persists after RNAi depletion of AMA-1, the large subunit of Pol II [8]. In this study we tested the transcription dependence of both MES-4 and MET-1 in embryos by treating wild-type, *mes-4* mutant, and *met-1* mutant worms with RNAi to *ama-1* and analyzing 80–110-cell embryos for H3K36me3. The persistence of some H3K36me3 after depletion of AMA-1 from wild-type suggests that H3K36me3 is at least partly Pol II-independent (Figure 1E). H3K36me3 staining was undetectable after simultaneous loss of MES-4 and AMA-1, but was easily detected after simultaneous loss of MET-1 and AMA-1 (Figure 1F and 1G). These results are consistent with MET-1 mediating Pol II-dependent H3K36me3, and indicate that MES-4 mediates at least some Pol II-independent H3K36me3. There are no precedents for Pol II-uncoupled H3K36me3. To further investigate the Pol II-independent binding and methylation of targets by MES-4, and to help understand how this activity promotes the viability of PGCs, we analyzed MES-4 binding across the genome at high resolution using ChIP-chip.

### Sites of MES-4 binding are distributed along the five autosomes and are under-represented on the X chromosome

**Figure 2:**
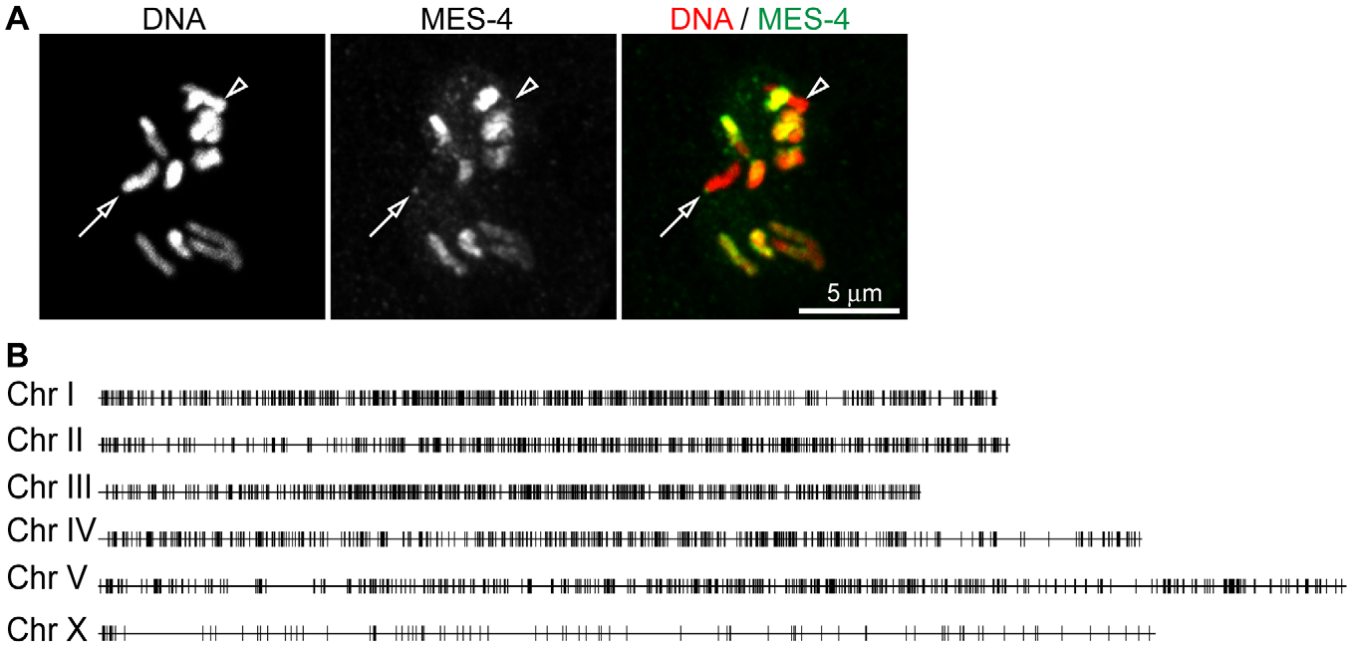
MES-4 is concentrated on the autosomes and the left tip of the X chromosome. (A) The anti-MES-4 antibody used for ChIP stains the 10 autosomes and the left tip of the X (arrow; the other X chromosome is marked with an arrowhead), similar to previously characterized antibodies [8], [19]. The MES-4 signal was over-exposed to show the dot of MES-4 on the left end of X and the absence of detectable MES-4 along the rest of the X. Additional staining, including absence of MES-4 signal in a *mes-4* mutant, is shown in Figure S2B. (B) 5291 of the 5391 MES-4 binding sites are distributed along the five autosomes. Of the 100 MES-4 peaks on the X chromosome, 25 are in the leftmost 300 kb. The expected and observed numbers of MES-4 peaks on each chromosome are in Figure S2D. All of the ChIPs shown in Figure 2, Figure 3, Figure 4, Figure 5, and Figure 6 were performed in early embryo extracts.

**Figure 3:**
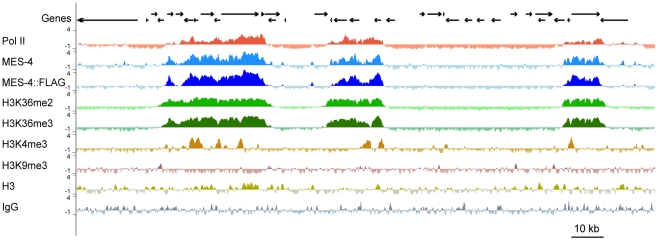
MES-4 is mostly concentrated on Pol II–bound genes. For a representative ∼180 kb autosomal (ChrIV) region of the genome, annotated genes (arrows; for genes encoding multiple transcript variants, the longest ones are shown) and ChIP z-scores (standardized log_2_ ratios of ChIP/Input signals) for Pol II, MES-4, MES-4::FLAG, H3K36me2, H3K36me3, H3K4me3, H3K9me3, H3, and a control ChIP (Protein A beads + rabbit IgG) are shown. Pol II, MES-4, H3K36me2 and H3K36me3 generally colocalize over bodies of transcribed genes. H3K4me3 is mainly found in the promoter and 5′ coding region of transcribed genes. H3K9me3 is absent from expressed genes. See Figure S3 for analysis of reproducibility and concordance among ChIPs.

To determine the distribution of MES-4 across the genome by ChIP-chip, we used rabbit anti-MES-4 antibody to immunoprecipitate endogenous MES-4 (Figure 2A and Figure S2A), and as an alternative strategy used a mouse anti-FLAG antibody to immunoprecipitate FLAG-tagged MES-4 (Figure S2C). Tagged MES-4 was judged to be functional by two criteria: the autosomal concentration of MES-4::GFP::FLAG resembles that of endogenous MES-4 (Figure S2), and the transgene rescues *mes-4(bn73)* mutant worms (see Text S1). ChIP was performed on extracts from early embryos for several reasons. First, MES-4 levels appear relatively low in the maternal germ line and dramatically increase after fertilization (Y. Fong and S. Strome, unpublished result), suggesting that early embryogenesis is a period important for MES-4 function and that ChIP signals may be more robust from early embryos than from isolated germline tissue. Second, MES-4 binding is concentrated on autosomes at all stages analyzed, including early embryos, suggesting that the mechanisms that control selective recruitment to the autosomes are operational in early embryos [19]. Third, early embryos can be harvested in quantities sufficient for ChIP, while pure germline tissue currently cannot. ChIP was performed in triplicate from sheared chromatin prepared from formaldehyde-fixed early embryos (see Materials and Methods). As controls, we performed ChIP using Protein A and Protein G beads and also beads coated with antibodies that were not specific to *C. elegans* proteins. Endogenous MES-4 and tagged MES-4 displayed remarkably concordant ChIP distributions, which differed significantly from those of controls (Figure 3 and Figure S3). Here, we focus on ChIP analysis of endogenous MES-4.

As observed by immunofluorescence staining of whole chromosomes, MES-4 ChIP peaks are concentrated on the autosomes and the left tip of the X. The peak-finding algorithm ChIPOTLe [31] (http://sourceforge.net/projects/chipotle-2/) was used to identify 5391 genomic regions with significant MES-4 enrichment. 5291 of the 5391 MES-4 binding sites are distributed along the five autosomes. Only 100 of the 5391 MES-4 sites (1.9%) are located on the X chromosome, in contrast to the 953 (17.7%) expected on the X if MES-4 were distributed uniformly across chromosomes (p-value<10^−131^) (Figure 2B and Figure S2D). 25% of MES-4 binding sites on the X are located within the leftmost 300 kb (1.7%) of the X. The density of binding sites on the left tip of the X (0.08 sites/kb) is similar to that on the autosomes (0.06 sites/kb). The remaining ∼17.4 Mb of the X chromosome have an extremely low density of MES-4 sites (0.004 sites/kb), which is apparently too low to detect by immunofluorescence. The far left tip of X, which resembles the autosomes in MES-4 binding density, is indeed marked by a dot of MES-4 immunostaining [8] (Figure 2).

### MES-4 associates with the coding regions of expressed genes

MES-4-bound regions generally overlie the coding regions of single genes and sets of adjacent genes (Figure 3). Of the 5391 MES-4 binding sites, 5217 overlap the coding region of at least 1 gene, 143 overlap the 1 kb upstream (64) or downstream (79) of at least 1 gene, and 31 reside in intergenic regions (Table S1A). Conversely, of the 22,241 annotated genes in WormBase, 4400 overlap a MES-4-bound site and an additional 98 overlap in their 1 kb upstream or downstream region. Thus, the large majority (>96%) of MES-4 overlies genic regions, and approximately 1 in 5 genes is associated with MES-4.

To investigate whether MES-4 generally accumulates on expressed or silent genes, we compared the distribution of MES-4 in early embryos to that of Pol II across the genome. ChIP analysis of Pol II was performed using the 8WG16 monoclonal antibody, which detects both unphosphorylated and phosphoryated forms of Pol II [32]. Most genes bound by MES-4 are also associated with Pol II. This is illustrated both by viewing individual genes (Figure 3) and by plotting MES-4 ChIP signal versus Pol II ChIP signal for all genes (Figure S4C). However, as discussed below, certain gene classes with specific temporal or spatial expression patterns have high MES-4 but no Pol II or high Pol II and no MES-4, indicating that Pol II is neither necessary nor sufficient to recruit MES-4.

**Figure 4:**
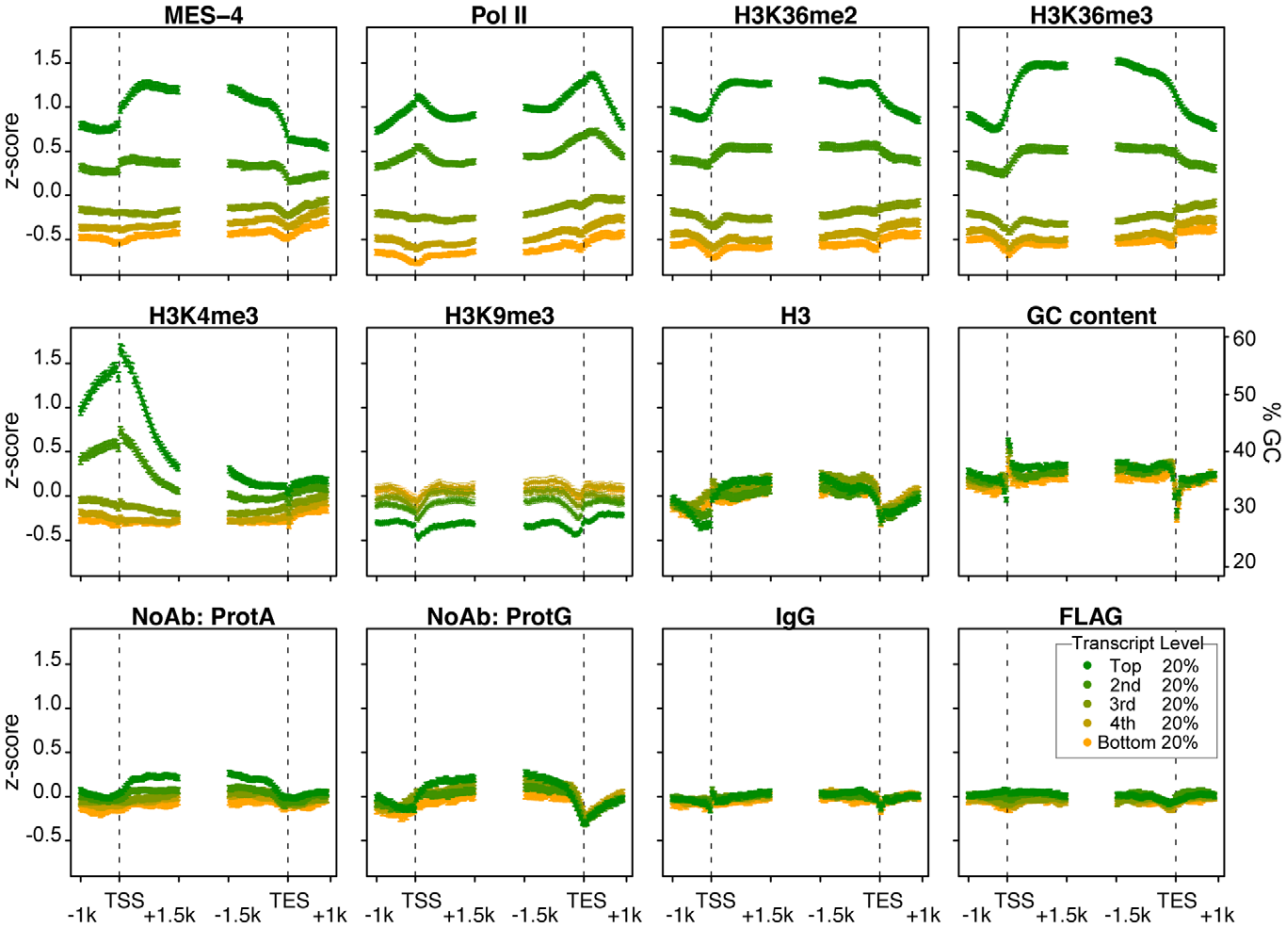
MES-4 and H3K36 methyl marks are concentrated over gene bodies of highly expressed genes. Genes >2 kb long (9932 genes) were grouped into 5 equal sized bins based on expression in early embryos. Colors display bins with highest (green) to lowest (orange) transcript level in early embryo extracts generated in parallel with ChIP extracts. Average z-score profiles for each ChIP target are shown in 50 bp steps in the 2.5 kb around the transcript start site (TSS) and the transcript end site (TES). Error bars indicate 95% confidence intervals for the means. The bottom row shows four different control ChIPs: Protein A beads, Protein G beads, Protein A beads coated with rabbit IgG, and sheep anti-mouse beads coated with mouse anti-FLAG. GC content variation is also shown.

Viewing the distribution of MES-4 and Pol II across gene bodies stratified by transcript accumulation in early embryos revealed that MES-4 levels are highest on the most highly transcribed genes and that MES-4 overlies gene bodies (Figure 4). Genes aligned at their transcript start sites (TSS) and end sites (TES) were binned into 5 equally sized groups based on microarray measurements of transcript levels in the extracts used for ChIP. Average MES-4 and Pol II profiles for genes within each bin were plotted starting from 1 kb upstream to 1.5 kb downstream of the TSS and from 1.5 kb upstream to 1 kb downstream of the TES (Figure 4). Genes with highest RNA level show the highest MES-4 binding, followed by progressively lower levels of MES-4 on genes with lower RNA levels. In the most highly expressed genes, MES-4 levels rise across the TSS, reaching a maximum value ∼500 bp into the gene body, gradually decline beyond that and drop at the TES. Pol II shows a different profile than MES-4. Pol II levels peak at the TSS and again 3′ of the TES (Figure 4). Although MES-4 and Pol II accumulate on mostly the same genes, the 3′ peak of Pol II compared to the 3′ drop of MES-4 suggests that MES-4 does not track with Pol II or that it tracks only with a subpopulation of Pol II.

### The methylation of H3K36 generally mirrors MES-4 accumulation

To compare the distribution of MES-4 to the distributions of H3K36 methyl marks in early embryos, we performed ChIP-chip using antibodies to H3K36me2 and H3K36me3. We also analyzed H3K4me3 and H3K9me3. All histone mark ChIPs were performed using highly specific monoclonal antibodies [33] (Figure S1).

The H3K36me2 and H3K36me3 ChIP profiles generally mirror those of MES-4. MES-4 and H3K36me2 and me3 ChIP signals on all genes are highly correlated (Figure 3 and ). In the bodies of actively expressed genes, H3K36me2 and me3 levels rise across the TSS and drop across the TES (Figure 4). The relatively flat profiles of H3K36me2 and me3 across gene bodies contrast with the 3′ enriched profiles of H3K36 methylation generated by Set2 homologs in yeast and other systems [e.g. 11], [25]. The distinctive gene-body profiles of H3K36me2 and me3 are not shared by other histone methyl marks. In keeping with H3K4me3 being a hallmark of active chromatin and specifically marking transcription initiation, H3K4me3 is distributed around the TSS, and declines sharply within gene bodies (Figure 4). H3K9me3, which is mostly associated with transcriptionally silent chromatin, indeed shows an inverse relationship to gene expression (Figure 4). The histone mark patterns described above can be compared to histone H3, which reflects nucleosome occupancy. We find that H3 is modestly elevated in gene bodies and shows a pronounced dip in the promoter region of highly expressed genes, as has been reported in other organisms [e.g. 25].

### Genes bound by MES-4 in early embryos are those that had been expressed previously in the maternal germ line

**Figure 5:**
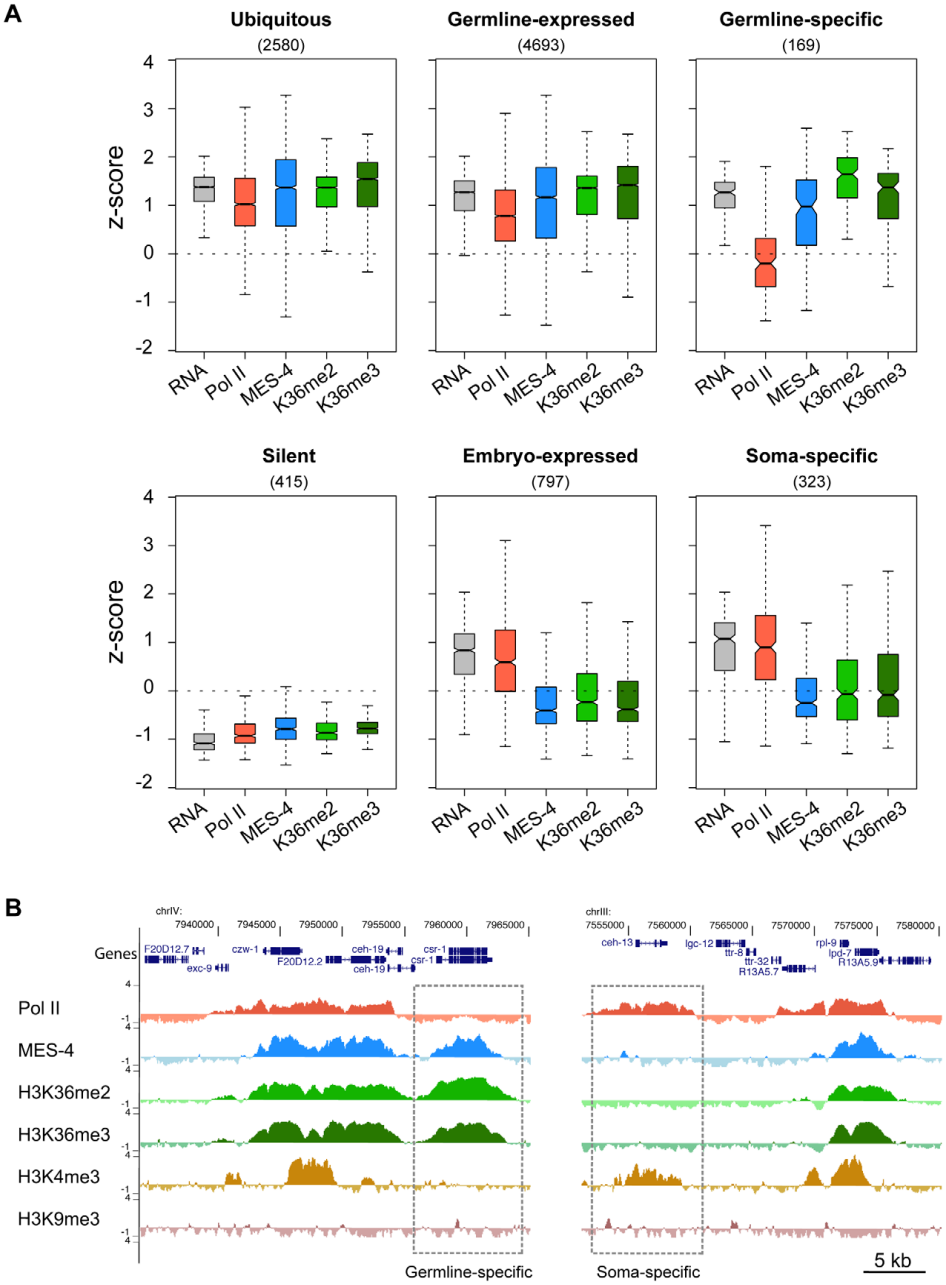
Distribution of RNA, Pol II, MES-4, H3K36me2, and H3K36me3 levels for various gene sets. (A) Gene classes were defined as described in Results. The number of genes in each set is shown in parentheses. For each gene set, boxplots show the distribution of RNA level (standardized to z-score calculated from our microarray analysis) and Pol II, MES-4, H3K36me2 and H3K36me3 average z-scores from early embryo ChIP-chip analysis. Each box extends from the 25^th^ to 75^th^ percentile of the z-scores in the set, and the whiskers extending from a box indicate the 2.5^th^ and 97.5^th^ percentile. Wedges around the median indicate 95% confidence interval for the medians. See Figure S4C for scatter plots of the ubiquitous, germline-expressed, germline-specific and embryo-expressed categories. (B) Examples of Pol II- MES-4+ H3K36me+ and Pol II+ MES-4− H3K36me− genes. The germline-expressed gene *csr-1* (left dashed box) contains high levels of MES-4, H3K36me2 and H3K36me3 but lacks Pol II and H3K4me3. The soma-expressed gene *ceh-13* (right dashed box) contains Pol II and H3K4me3 but lacks MES-4, H3K36me2 and H3K36me3. Both *csr-1* and *ceh-13* are flanked by genes that show the typical high concordance between Pol II, MES-4 and H3K36me marks. See Table S2 for lists of genes in different temporal-spatial classes and Figure 6C for more examples of individual genes.

MES-4 is essential for germline development and survival, and knowing which genes are bound by MES-4 is critical to understanding its mechanism of action. Gene Ontology (GO) analysis of all MES-4-bound genes revealed that MES-4 preferentially associates with genes that participate in reproduction, growth and development (Table S1C). To further characterize MES-4 target genes, we analyzed RNA levels in conjunction with MES-4, Pol II, and H3K36me2 and me3 ChIP signals on genes that exhibit different temporal-spatial expression patterns (Figure 5A). We generated six gene categories, based on previous studies (Table S2): (1) 4693 “germline-expressed” genes whose transcripts are present in dissected adult hermaphrodite germ lines according to SAGE analysis [34]; (2) 169 “germline-specific” genes whose transcripts are expressed exclusively in the maternal germ line (transcripts were detected in dissected hermaphrodite germ lines by SAGE, are enriched in the germ line based on whole-worm microarrays, accumulate in embryos strictly by maternal contribution as determined by microarray analysis of staged embryos, and are not represented in muscle, gut, or neuron SAGE sets) [21], [34]–[36]; (3) 797 “embryo-expressed” genes whose transcripts are not maternally provided and increase in level during embryogenesis [35]; (4) 323 “soma-specific” genes whose transcripts are detected in muscle, gut, and neuron SAGE sets but not in the adult germ line by SAGE or microarray analysis [21], [34], [36]; (5) 2580 “ubiquitous” or housekeeping genes whose transcripts are shared by muscle, gut, neuron and adult germline SAGE sets [34], [36]; and (6) 415 “silent” genes, serpentine receptor genes that are expressed in a few mature neurons and are not expected to be expressed in cells of early embryos [37]. The distinctive patterns of MES-4, Pol II, and H3K36 methylation on genes in different temporal-spatial classes provided key insights into MES-4 recruitment and function.

First, the two largest classes of genes, germline-expressed genes and ubiquitously-expressed genes, share a pattern of high RNA accumulation and high Pol II, MES-4 and H3K36me2/me3 association (Figure 5A and Figure S4C). The common theme among these genes is that they are expressed in the maternal germ line. Thus, in early embryo extracts, genes expressed in the maternal germ line typically remain bound by Pol II, MES-4, and H3K36me2/me3. As an interesting sidelight, the clustering of Pol II and MES-4-bound genes displayed in Figure 3 and observed across all autosomes is consistent with the finding that ∼40% of genes with germline-enriched expression are organized in operons, and that those operons further cluster with monocistronic germline-expressed genes [38].

Second, silent genes exhibit low RNA accumulation and low Pol II, MES-4, and H3K36me2/me3 (Figure 5A). This result fits with seeing lowest levels of Pol II, MES-4, and H3K36me2/me3 association with genes in the bottom quantiles of expression (Figure 4).

**Figure 6:**
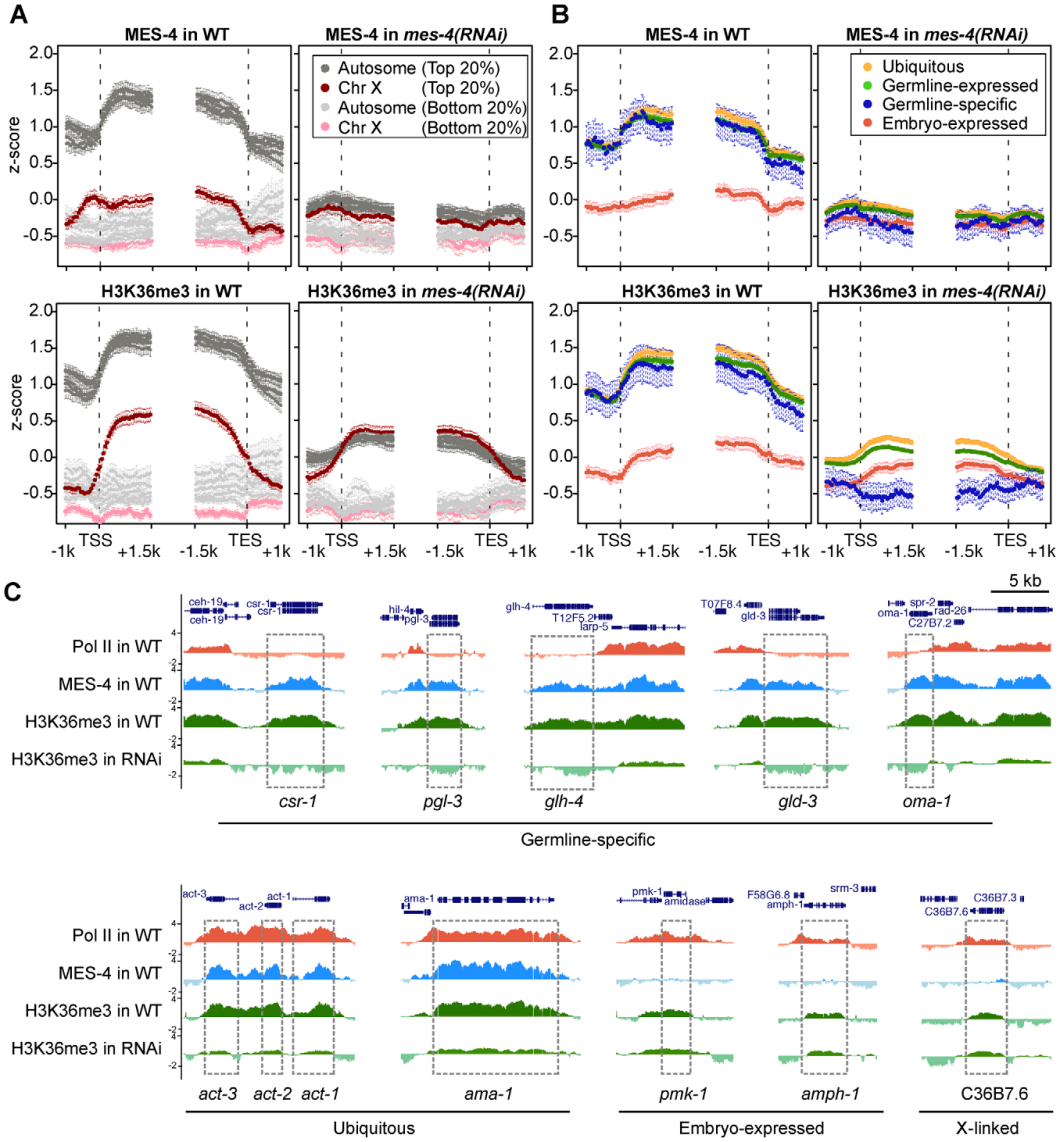
RNAi depletion of MES-4 substantially reduces H3K36me3 on germline-expressed genes. (A) MES-4 and H3K36me3 ChIP-chip signal on the autosomes (gray) and the X chromosome (red), specifically on genes with the highest expression in wild type (top 20% in dark color) and genes with the lowest expression in wild type (lowest 20% in light color). MES-4 is dramatically reduced genome-wide in *mes-4(RNAi)*. H3K36me3 on autosomes is reduced to the lower level observed on the X, from which MES-4 is largely absent in wild type. The remaining low level of H3K36me3 on transcribed genes in *mes-4(RNAi)* is most likely due to transcription-coupled methylation by MET-1. (B) MES-4 and H3K36me3 ChIP-chip signal on ubiquitously-expressed (orange), germline-expressed (green), germline-specific (blue), and embryo-expressed (red) gene sets in wild type and *mes-4(RNAi)*. In *mes-4(RNAi)*, H3K36me3 is dramatically reduced on genes expressed in the germline (ubiquitous and germline-expressed) and undetectable on germline-specific genes. The embryo-expressed class (and soma-specific class, not shown) lack MES-4 in wild type and show little change in *mes-4(RNAi)*. (C) Examples of individual genes illustrating the patterns observed in (B).

Third and most importantly, the germline-specific class shows a distinct pattern: high RNA accumulation, high MES-4 and H3K36me2/me3 signals, but low Pol II (Figure 5A and Figure S4C). To explore this deviation from the usual high correlation between MES-4 and Pol II, we analyzed individual germline-specific genes in the UCSC genome browser. Numerous genes displayed robust MES-4 and H3K36me2/me3 ChIP signal but no detectable Pol II ChIP signal (Figure 5B and Figure 6C). We identified 214 genes as having high MES-4 and high H3K36me3 (z-scores>1) and low Pol II (z-score<0) (Table S3). Notably, 180 of the 214 genes (84%) show evidence of germline expression based on microarray and/or SAGE analysis [21], [34], and many of those genes are known to be involved in germline-specific processes (e.g. the meiosis genes *him-8*, *htp-1*, *htp-2*, *htp-3*, *rec-8*, *syp-2*, *zim-2*, *zim-3*, and the P-granule genes *pgl-1*, *pgl-3*, *glh-2*, *glh-4*). The germline-specific class of genes indicates that MES-4 can persist on genes that were previously expressed in the maternal germ line but are no longer being actively transcribed in early embryos, revealing that MES-4 can associate with genes independently of Pol II. This class probably contributes to the MES-4 and H3K36me2 and me3 staining observed after depletion of Pol II (Figure 1E) [8].

Fourth, embryonically-expressed genes and soma-specific genes are silent in the maternal germ line but must be activated during embryogenesis. These genes display the unusual pattern of high RNA accumulation and high Pol II but low MES-4 and H3K36me2/me3 (Figure 5A and Figure S4C). In fact, inspection of individual genes in this class identified examples of robust Pol II ChIP signal but low or no detectable MES-4 or H3K36me2/me3 (Figure 5B, Figure 6C, and Figure S4C). These findings suggest that although MES-4 is found mostly on Pol II-bound genes (the first two large classes discussed above) (Figure 3, Figure S3B, and Figure S4C), Pol II accumulation is not sufficient to recruit MES-4.

In addition to clues about MES-4, some members of the embryonically-expressed and soma-specific genes provide the first natural examples of transcription elongation in the absence of detectable H3K36 methylation (Figure 5B and Figure S4C), a phenomenon observed only in yeast *set2* mutants previously [9]. The *C. elegans* Set2 ortholog MET-1, which participates in transcription-dependent H3K36 methylation in embryos (Figure 1), is expected to methylate H3K36 on genes newly expressed in embryos. By immunofluorescence, MET-1-mediated H3K36me3 is not detected in 2- to 40-cell embryos and gradually rises from the 40-cell stage onward (Furuhashi et al., unpublished and our unpublished results). In our ChIP experiments, genes bound by Pol II but lacking MES-4 display little or no H3K36me2/me3 (Figure S4C). Thus, in our early embryo samples, MET-1 appears to be a relatively minor contributor to H3K36 methylation.

### MES-4 is responsible for H3K36 methylation of germline-specific genes

The above results strongly suggest that MES-4 mediates the majority of H3K36 methylation in early embryos. To investigate MES-4's contribution, we depleted MES-4 by RNAi and performed ChIP-chip analysis of MES-4 and H3K36me3. Evidence that RNAi was effective was provided by the following observations: the *mes-4(RNAi)* embryos used for ChIP lacked detectable MES-4 immunostaining, and MES-4 and H3K36me3 ChIP signal on autosomes, where MES-4 overwhelmingly resides in wild type, was reduced in *mes-4(RNAi)* extract to the low level observed on the X chromosome (Figure 6A). Importantly, H3K36me3 ChIP signal in *mes-4(RNAi)* was completely lost from germline-specific genes (Figure 6B and 6C), supporting our hypothesis that these genes are H3K36 methylated solely by MES-4 and in the absence of active transcription. H3K36me3 signal on ubiquitously-expressed and germline-expressed genes, the majority of which are actively transcribed in early embryos, was reduced but not eliminated (Figure 6B and 6C). The remaining H3K36 methylation of these genes is probably due to (low) MET-1 activity and possibly incomplete knock-down of MES-4. Embryo-expressed and soma-specific genes showed little change in H3K36me3 levels in *mes-4(RNAi)*, many retaining the low levels of H3K36me3 seen in wild type (Figure 6B and 6C). This again suggests that detection of H3K36me3 is due to low MET-1 activity on this set of actively transcribed genes. Taken together, our RNAi results demonstrate that during embryogenesis MES-4 is responsible for H3K36 trimethylation of germline-specific genes and contributes the majority of H3K36 trimethylation on other germline-expressed genes as well.

### The RNA polymerase-independent epigenetic function of MES-4 is reflected in the distribution of H3K36me3 on target genes

**Figure 7:**
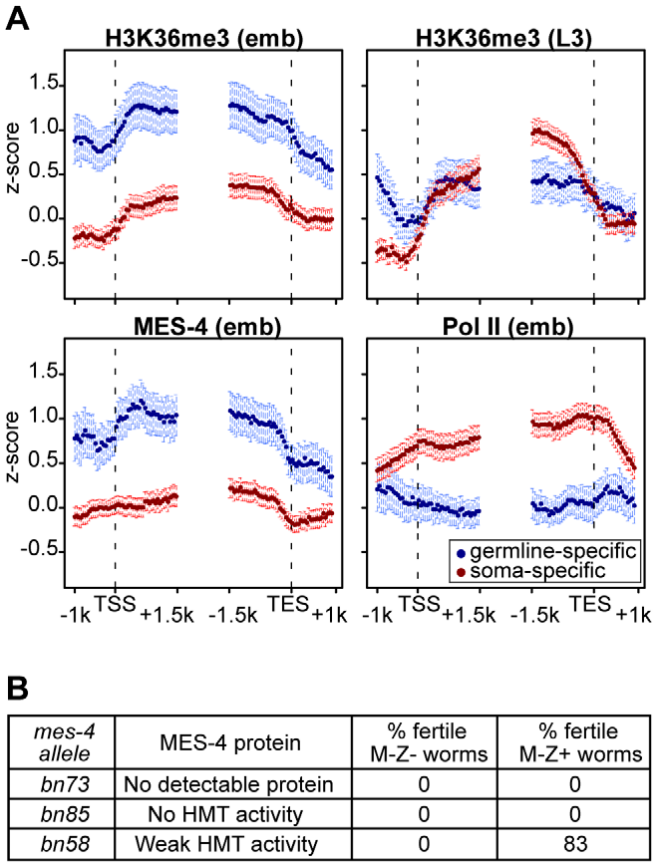
The distribution of and requirement for MES-4-catalyzed H3K36 methyl marks. (A) Germline-specific and soma-specific genes display different profiles of H3K36me3, MES-4, and Pol II in early embryos (emb) and H3K36me3 in L3 larvae (L3 data are from [37]). Gene profiles are as described for Figure 4, using the germline- and soma-specific genes described in Results. This figure highlights the reciprocal relationship between Pol II levels and MES-4/H3K36me3 levels on germline- and soma-specific genes in early embryos (also see Figure 5). On germline-specific genes, H3K36me3, like MES-4, is slightly more elevated in 5′ than 3′ gene bodies, whereas on soma-specific genes, H3K36me3 is more elevated in 3′ than 5′ gene bodies, especially in L3s, probably due to transcription-dependent methylation by MET-1. (B) Embryonic expression of MES-4(+) rescues fertility of *mes-4* mutants only when maternal MES-4 retains some HMT activity. Analysis of mutant MES-4 proteins is from [8]. M-Z− (lacking both maternal and zygotic expression of *mes-4*+) worms were *mes-4/mes-4* hermaphrodites produced by *mes-4/mes-4* mothers. M-Z+ worms were *mes-4*/+ hermaphrodites produced by mating *mes-4/mes-4* mothers with wild-type males. At least 24 M-Z+ hermaphrodites were assessed for fertility. 75% of *bn58*/+ hermaphrodites produced viable progeny; 8% produced embryos that failed to hatch.

The above results reveal that in embryos MES-4 can associate with genes and maintain H3K36 methylation in a Pol II-independent manner. If MET-1 resembles Set2 in being recruited to genes by elongating Pol II, then MET-1-generated H3K36me3 should be enriched in the 3′ coding region of genes, similar to H3K36me3 generated by other known Set2 homologs [11], [23], [25], [39]. Indeed, soma-specific genes, which generally lack MES-4 binding and are presumed to acquire H3K36 methylation from MET-1, display such an enrichment in the 3′ gene body (Figure 7A in red). This pattern is relatively low-level in early embryos in which MET-1 activity is low (see discussion in previous section) and more pronounced in later stages (L3 larvae; data from [37]) in which somatic cells are actively engaged in transcription and MET-1 level and activity are expected to be elevated. In contrast, germline-specific genes display a distribution of H3K36me3 that is slightly 5′ enriched, similar to the distribution of MES-4 (Figure 7A in blue). The difference in H3K36me3 profiles on germline- versus soma-specific genes strengthens the notion that MES-4 and MET-1 are recruited to genes by different mechanisms and serve different roles on their targets. We hypothesize that MES-4 serves an epigenetic role and transmits the memory of gene expression in the adult germ line to cells of the embryo (see Discussion).

### The H3K36 methyltransferase activity of maternal MES-4 is crucial for germline development in progeny

Embryos from *mes-4/mes-4* mothers develop into sterile adults, and embryonic expression of MES-4(+) is not sufficient to restore fertility [18]. Based on our findings that in embryos MES-4 persists on germline-expressed genes even in the absence of ongoing transcription, we reasoned that the failure of embryonic expression of MES-4(+) to rescue fertility was likely due to loss of H3K36 methylation generated by maternal MES-4(+) and an inability of embryonically expressed MES-4(+) to newly generate the patterns of H3K36 methylation needed for normal PGC development. Previous tests for rescue of fertility by embryonic expression of MES-4(+) were performed using *mes-4/mes-4* mutant mothers that lack detectable H3K36 HMT activity (*bn23*, *bn50*, and *bn67* alleles of *mes-4* [8], [18]). We tested several additional alleles with the goal of assessing whether *mes-4/mes-4* mutant mothers with weak HMT activity would provide sufficient H3K36 marking to enable embryonic expression of MES-4(+) to rescue fertility. The *bn73* allele does not produce detectable MES-4 protein, the *bn85* allele produces MES-4 lacking part of the catalytic SET domain, and the *bn58* allele produces MES-4 with weak HMT activity [8]. Embryonic expression of MES-4(+) did not restore fertility to animals from MES-4(−) mothers (*bn73* allele) or from mothers that produce MES-4 lacking HMT activity (*bn85* allele) (Figure 7B). Importantly, embryonic expression of MES-4(+) restored fertility to animals whose mothers produced mutant MES-4 with weak HMT activity (*bn58* allele) (Figure 7B). This demonstrates that the HMT activity of MES-4 is critical for maternal MES-4 function, and strengthens the view that MES-4 acts epigenetically and transmits developmentally important H3K36 marks from the parents' germ line to embryos, to ensure normal development of PGCs.

## Discussion

MES-4 has emerged as a critical germline regulator in several studies. Its maternal-effect sterile (mes) phenotype demonstrates that the action of MES-4(+) in the maternal germ line and/or a maternal supply of MES-4(+) is required for the PGCs in offspring to thrive and survive [18]. Germline functions that require MES-4 are repression of genes on the X chromosome, transgene silencing, cosuppression, and RNAi [8], [40]–[42]. In addition to all of these repressive roles, MES-4 is required to promote ectopic expression of germline genes in somatic cells of synMuv B mutant larvae [43], [44]. The current study identified MES-4 binding targets as being genes expressed in the adult germ line. The targeting and function of MES-4 appear to be distinct from the well-studied Set2 family of H3K36 HMTs. We speculate that MES-4 transmits the memory of germline gene expression patterns from the parental germ line to the PGCs in offspring, and that loss of this function causes PGCs to degenerate in young larvae.

By both immunofluorescence and ChIP-chip analysis, MES-4 is concentrated on the five autosomes and the very left tip of the X ([8], [19] and this study). The strong autosomal bias of MES-4 ChIP signal is not due to an X chromosome structure that is recalcitrant to ChIP, as ChIP analysis of the dosage compensation complex preferentially targets the X [45], [46]. Instead, the autosomal enrichment of MES-4 is likely explained by the association of MES-4 with germline-expressed genes, which are significantly under-represented on the X [21]. The left end of X has several distinquishing properties, including a relatively high concentration of H3K9me3 and low density of periodic AA/TT clusters compared to the rest of the X [47] and the presence of the X chromosome “pairing center” for meiosis [48]. The autosome-like higher density of MES-4 binding sites on the left end of X is consistent with its being “pseudo-autosomal” in nature. Perhaps it arose by translocation of an autosomal segment to the end of the X. In fact, comparison of the genomes of *C. elegans* and *Pristionchus pacificus* suggests that they evolved from a common ancestor in which chromosomes X and V were fused ([49] and R. Sommer, personnal communication). The autosomal concentration of MES-4 is regulated by the other three MES proteins, MES-2, MES-3, and MES-6, which form the *C. elegans* Polycomb Repressive Complex 2 and like PRC2 catalyze methylation of H3K27 [50]–[52]. Loss of any component of the MES-2/3/6 complex leads to loss of silencing of the X chromosomes in the germ line and significant MES-4 immunofluorescence signal along the full length of the X in oocytes and embryos [8], [19]. Our model predicts that in *mes-2*, *mes-3*, *and mes-6* mutant embryos, elevated MES-4 signal will be observed on X-linked genes whose expression was up-regulated in the maternal germ line.

Our studies demonstrate that in early embryos MES-4 associates with genes that were expressed in the maternal germ line. Genes expressed exclusively in the maternal germ line have high MES-4 and low Pol II. Some individual genes are MES-4+ Pol II−. These genes strongly argue that Pol II is not *required* to maintain MES-4 association with genes. Conversely, genes newly expressed in embryos and genes expressed specifically in somatic cells have high Pol II and low MES-4. Indeed, numerous individual genes are Pol II+ MES-4−. This is consistent with Pol II not being *sufficient* to recruit MES-4 to expressed genes.

Additional evidence supports the notion that MES-4 is capable of associating with gene bodies and methylating H3K36 independently of Pol II. First, RNAi depletion of the large subunit of RNA Pol II does not impair MES-4 binding to chromosomes or H3K36 di− or trimethylation, as detected by immunofluorescence ([8] and this study). Second, the slightly 5′ enriched distribution of MES-4 across genes bodies is quite different from the 3′ enriched distribution of Set2 homologs, the latter driven by association of Set2 with elongating Pol II [11], [23], [25], [39]. Recent studies in yeast have revealed that Set2 can be recruited to genes independently of Pol II, but is impaired in H3K36 di− and trimethylation [53]. In contrast, MES-4 appears capable of di− and trimethylating H3K36 in the absence of Pol II. Taken together, our findings suggest that MES-4 and perhaps the related mammalian NSD proteins provide another layer of function for H3K36 methylation that is novel and likely to be unrelated to ongoing transcription.

An attractive model is that MES-4 serves as a maintenance HMT to mark germline-expressed genes and pass the memory of gene expression from one generation of germ cells to the next. In this model, the H3K36 HMT MET-1 serves a Set2-like role and tracks with Pol II to methylate H3K36 during gene expression in the adult germ line; during embryogenesis, MES-4 maintains H3K36 methylation on those genes independently of Pol II. We think that MES-4 can serve this maintenance role even in transcriptionally repressed cells (e.g. in the germline blastomeres) and potentially for generations (e.g. in *met-1* mutant worms). In support of this model, MES-4 does not appear to be capable of de novo H3K36 methylation in the soma and embryonic PGCs: embryonic expression of MES-4 in embryos that lack maternal H3K36me3 (*mes-4*; *met-1* double mutant mothers) does not generate detectable H3K36me3 signal (Furuhashi et al., unpublished). In contrast, embryonic expression of MET-1 generates robust H3K36me3. Thus, our model posits that MES-4 is a specialized maintenance HMT that in embryos can only methylate chromatin with pre-existing H3K36 methyl marks. Consistent with this, embryonic expression of MES-4(+) can rescue the fertility of embryos from mothers that produce mutant MES-4 with weak HMT activity, but not embryos from mothers that lack MES-4 or that produce MES-4 that lacks HMT activity. The maintenance HMT activity of MES-4 enables MES-4 to serve a truly epigenetic role, propagation of a particular chromatin state through meiosis and mitosis. Recent studies demonstrate that the Polycomb Repressive Complex 2 both initiates and maintains a repressed chromatin state, the latter by binding the chromatin marks that it generates [6].

How is MES-4 initially recruited to germline-expressed genes? Our findings that MES-4 can associate with genes and generate H3K36 methylation independently of Pol II do not rule out the possibility that MES-4 has a Pol II-dependent mode as well. In the adult germ line, MES-4 may be initially recruited to expressed genes in a Pol II-dependent manner. Another possibility is that MES-4, perhaps via its PHD fingers, binds H3K36 methyl marks generated by MET-1 and/or MES-4. Yet a third possibility is that the chromodomain protein MRG-1, like its counterparts in other systems [12], [27], [28], [30], [54], associates with methylated H3K36 and helps recruit MES-4. In fact, MRG-1, like MES-4, displays autosomal enrichment by immunofluorescence, and *mrg-1* mutants display a suite of *mes-4*-like defects, including maternal-effect death of PGCs [55]. MES-4 association with autosomes is not lost in *mrg-1* mutants and vice versa [55]. Determining whether MRG-1 participates in recruiting MES-4 and/or is a downstream effector of MES-4-mediated H3K36 methylation is in progress.

An important question for future investigation is why the PGCs in embryos from *mes-4* mutant mothers die. An attractive scenario is that MES-4 marking of genes expressed in the maternal germ line identifies genes to be expressed in the progeny's germ line. A related scenario, which might temporally precede the first, is that MES-4 marking of genes helps keep those genes repressed in the PGCs during embryogenesis. Indeed, wild-type PGCs generally do not acquire marks of active transcription until after embryos hatch into L1s, while *mes-4* mutant PGCs acquire such marks prematurely, during mid-embryogenesis ([56] and Furuhashi et al., unpublished). In addition to a potential role in up- or down-regulating transcription in the PGCs, MES-4 may influence gene expression at the level of regulation of splicing. Recent papers report that H3K36me3 is enriched in expressed exons relative to introns [37] and that the level of H3K36me3 influences alternative splicing [57]. Like H3K36me3, MES-4 appears to be exon-enriched ( and Figure S5), raising the exciting possibility that MES-4 preferentially associates with exons and methylates them in a manner that facilitates or regulates splicing. Experiments are planned to isolate PGCs from wild-type and *mes-4* mutant embryos and compare their RNA accumulation and splicing patterns. In the meantime, we analyzed the overlap between MES-4-bound genes in embryos and genes mis-regulated in *mes-4* mutant adult germ lines, and found it to be small (Figure S6). The small overlap may be due to the stage difference (i.e. embryo versus adult germ line) or to technical and biological effects that cause even well documented transcription regulators to display a low overlap between factor-bound genes and genes mis-regulated when the factor is absent [e.g. 58], [59]. Despite the absence of gene expression data in PGCs, the strong dependence of early PGC development on maternal MES-4 demonstrates the functional importance of MES-4.

MES-4 and its HMT activity are detected in all cells of early embryos and become restricted to the PGCs during mid to late embryogenesis ([19] and our unpublished results). Our model that MES-4 propagates the memory of germline gene expression through embryogenesis raises the question how somatic cells in the embryo deal with that signal. Our current view is that the synMuv B chromatin regulators antagonize germline fate in somatic cells. Loss of synMuv B proteins causes somatic cells to express germline-specific genes, and concomitant loss of maternal MES-4 suppresses the germline potential of somatic cells [43], [44]. Further tests of our model and of the interplay between MES-4, MET-1, MRG-1 and the synMuv B chromatin regulators will shed light on how germline identity is passed from generation to generation and how germline gene expression patterns are controlled.

## Materials and Methods

### RNAi depletion of RNA polymerase II

We depleted AMA-1 by feeding N2 worms bacteria expressing dsRNA against *ama-1* (from the Ahringer RNAi feeding library [60]). In brief, L3-L4 hermaphrodites were fed for 24 hours at 24°C, then transferred to new plates to score the embryonic lethality of F1 progeny (>98%) or dissected to obtain embryos for staining.

### Generation of MES-4 ChIP reagents

Affinity purified rabbit antibodies directed against amino acids 729–828 of MES-4 were generated by Strategic Diagnostic Inc. (SDI, Newark, DE). *mes-4* animals expressing a MES-4::GFP::FLAG transgene were generated as described in Supplement.

### Preparation of N2 and *mes-4(RNAi)* early embryo extracts and chromatin

For preparation of embryo extracts, N2 adult worms were grown from synchronized L1s in standard S-basal medium with shaking at 230 rpm. N2 worms were fed HB101 for 58–60 hr at 20°C, or fed bacteria expressing dsRNA against *mes-4* (from the Ahringer RNAi feeding library [60]) for 48 hr at 24°C. To ensure that a majority of embryos used for extract preparation were early stages, worms were processed for extract only if the majority of worms contained 10 or fewer embryos. Embryos were obtained by dissolving adult worms with bleach and analyzed by DAPI staining. Embryo preparations contained an average of 37% <28-cell embryos, 30% 28–100-cell embryos, and 33% ∼100–300-cell embryos. 50 µl of packed embryos were set aside for RNA extraction and expression profiling. The remaining embryos were washed and cross-linked with 1.85% formaldehyde in M9 buffer for 30 min at room temperature, then washed for 5 min in each of the following buffers: twice with M9, once with 100 mM Tris-HCl pH7.5, once with 10 ml FA buffer (50 mM HEPES/KOH pH7.5, 1 mM EDTA, 1% Triton X-100, 0.1% sodium deoxycholate, 150 mM NaCl). Embryos were frozen at −80°C. 2 ml of embryos, thawed and resuspended in 4 ml of FA buffer containing protease inhibitors (Roche protease inhibitor cocktail tablet), were dounce-homogenized 30 times using a tight pestle at 4°C. Chromatin was sheared to an approximate size range of 200–800 bp using a Branson sonicator or a Diagenode bioruptor. To remove cellular debris, samples were centrifuged at 13,000 rpm, 4°C for 15 min. To remove lipids, samples were filtered through Millipore Ultrafree-MC 0.45 µm filter units at 13,000 rpm, 4°C for 1 min. Protein concentration was determined and samples were stored at −80°C.

### Chromatin immunoprecipitation (ChIP) from early embryos

ChIPs were performed using 1–6 mg of worm embryo protein and 2–5 µg antibody per IP. 5–10% of starting material was reserved as input. Rabbit antibodies used for IP were anti-histone H3 serum (Active Motif AR-0144) and affinity-purified anti-MES-4 (SDI). Mouse monoclonal antibodies used for IP were M2 anti-FLAG (Sigma F3165), 8WG16 anti-Pol II (Abcam ab817), anti-H3K4me3 (Wako 305-34819), anti-H3K9me3 (from Hiroshi Kimura), anti-H3K36me2 (from Hiroshi Kimura), and anti-H3K36me3 (from Hiroshi Kimura).

MES-4 and Pol II were ChIPed using 3 mg embryo protein and 5 µg antibody. For each ChIP reaction, antibody was incubated overnight at 4°C with embryo extract. Protein A- or Protein G-coupled Dynabeads (Invitrogen) equilibrated in 50 µl FA buffer were added, nutated for 2 hr at 4°C, concentrated using a Dynal Magnetic Particle Concentrator (MPC) (Invitrogen) and washed at room temperature for 5 min in 1 ml of each of the following buffers: FA buffer, 2 washes; FA buffer with 1M NaCl, 1 wash. Beads were transferred to a new tube, washed once for 10 min at room temperature in 1 ml FA buffer with 500 mM NaCl, once with TEL buffer (0.25 M LiCl, 1% NP-40, 1% sodium deoxycholate, 1 mM EDTA, 10 mM Tris-HCl, pH 8.0) and twice for 5 min at room temperature in 1 ml TE. To elute complexes, beads were incubated twice at 65°C in 150 µl of elution buffer (1% SDS in TE with 250 mM NaCl) for 15 min. Input samples were brought to a volume of 300 µl and incubated with 20 µg of RNAse A for 30 minutes at 37°C. Both input and IP samples were incubated with 20 µg of proteinase K for 1 hr at 55°C. Crosslinks were reversed overnight at 65°C. DNA was purified using a Zymo DNA purification column.

Histone H3 and methyl marks were ChIPed using 1 mg embryo protein and 2 µg antibody and the same method as above, except IP beads were Dynabeads coupled to sheep anti-mouse IgG (Invitrogen) equilibrated in 50 µl of 5 mg/ml IgG-free BSA (Sigma) in FA buffer, and both input and IP samples were incubated with RNAse A before proteinase K.

### Amplification by LM–PCR

Input and IPed DNAs were blunted with T4 DNA polymerase, purified using a Zymo DNA purification column, and ligated to a unidirectional linker prepared from the following HPLC-purified oligos: Long: 5′-AGAAGC TTGAATTCGAGCAGTCAG-3′ and Short: 5′-CTGCTCGAATTCAAGCTTCT-3′. DNAs were purified using a Zymo DNA purification column and amplified with the Short oligo in an 80 µl volume using the following cycling conditions: 55°C for 4 min, 72°C for 5 min, 94°C for 5 min followed by 25 cycles of: 94°C for 1 min, 55°C for 1 min, 72°C for 1 min. Samples were incubated for an additional 5 min at 72°C. DNA was purified using a Qiagen PCR purification kit and eluted in 30 or 50 µl dH_2_O.

### Microarray hybridizations and data analysis and display

NimbleGen 2.1M probe tiling arrays, with 50 bp probes, designed against WS170 (ce4) were used. 2–3 independent ChIPs were performed with each antibody and for each no antibody control (NoAb). Amplified samples were labeled and hybridized by the Roche NimbleGen Service Laboratory. Most ChIP samples were labeled with Cy5 and their input reference with Cy3; for most targets, one ChIP per antibody was dye swapped. For each probe, the intensity from the sample channel was divided by the reference channel and transformed to log_2_ space. The enrichment scores for each replicate were calculated by standardizing the log ratios to mean zero and standard deviation one (z-score). The *mes-4(RNAi)* ChIP samples were normalized with respect to the X chromosome in wild type. MES-4 was found to be largely absent from the X in wild type, and therefore was expected to be affected the least by RNAi. The log_2_ ratios for MES-4 and H3K36me3 in *mes-4(RNAi)* were normalized so that the X chromosome had the same mean and standard deviation as the X chromosome in the respective wild-type ChIP samples (after the wild-type ChIPs had been z-score normalized). Genome-wide Pearson correlations were calculated between all ChIP targets and replicates using all probes' z-scores after smoothing over 250 bp. The average z-score across replicates was used for all analyses.

Scatter plots and boxplots were generated by first obtaining an average z-score per gene. Z-scores of probes located completely within the transcript start site (TSS) and end site (TES) were averaged for each gene.

Gene body profile plots for the various ChIP targets were generated by aligning genes of length greater than 2 kb at their TSS and TES. The genomic regions 1.5 kb upstream to 1 kb downstream from the TSS and 1.5 kb upstream to 1 kb downstream from the TES were divided into 50 50-bp bins each. Probes in those genomic regions were assigned to the nearest bin. A profile for a group of genes was generated by averaging probes' z-scores within each bin across genes in the group. Error bars indicate 95% confidence intervals for the mean.

### ChiPOTle peak finding and analysis

ChiPOTle 2.0 (http://sourceforge.net/projects/chipotle-2) [31] was run using a p-value cut-off of E-20 for the average z-score of MES-4 and the Protein A NoAb control (window size 500 bp, step size 100 bp, Bonferroni p-value correction). 5408 peaks were identified for MES-4 and 38 for the NoAb control. 17 MES-4 peaks that were within 1 step (100 bp) of a NoAb peak were eliminated, producing the final set of 5391 MES-4 peaks. Expected values for number of peaks on different chromosomes were calculated by dividing the total number of peaks by chromosome length (Figure S2D).

### Peak-gene annotation analysis

The list of gene coordinates (transcript start-end) was downloaded from WormBase (http://www.wormbase.org/). We analyzed MES-4 peaks with respect to genomic annotations in three ways. First, MES-4 peaks that overlap with a gene by at least 1 bp in the gene body or within 1 kb of the gene's 5′ or 3′ were identified (Table S1A). Second, since one MES-4 peak could overlap with multiple genes, genes that overlap with a MES-4 peak by at least 1 bp within the gene body were identified, producing a set of “MES-4-bound” genes. Third, we determined the distribution of MES-4 peaks among different genomic annotations using Cis-regulatory Element Annotation System (CEAS) [61]. In CEAS, each peak is assigned to a single coordinate and using WormBase version WS170 the location of this coordinate with respect to the annotation classes exon, intron, 5′, 3′, and other (in our case >1 kb away) is determined. We chose the probe with the maximum enrichment value as a peak's coordinate. One coordinate can be assigned to multiple genomic annotations. Therefore, to assign a peak to a single region, we imposed the following hierarchy: exon, intron, 3′, 5′, 1 kb away. For comparison, all probes within the microarray were run through CEAS with the same parameters. Table S1B reports the results of MES-4 peak assignments to exons and introns.

### Transcription profiling from early embryos

RNA was isolated from four of the early embryo preparations used for ChIP using Trizol and purified using the RNeasy kit (Qiagen, catalog 74104). Samples were analyzed on an Agilent Bioanalyzer to ensure that rRNAs were not degraded and that RNA was free of protein and DNA contamination. Two of the samples were treated with DNase for 30 min at room temperature. 20 µg of each RNA were hybridized to a single color 4-plex NimbleGen expression array with 72,000 probes (three 60-mer oligo probes per gene). Quantile normalization [62] and the Robust Multichip Average (RMA) algorithm [63] were used to normalize and summarize the multiple probe values per gene to obtain one expression value per gene and sample. The expression values per gene were averaged across the four samples.

### Assessment of rescue by embryonic expression of MES-4(+)

*mes-4(bn58, bn73, or bn85) dpy-11(e224)/DnT1[unc(n754)let] (IV;V)* mothers were allowed to produce *mes-4 dpy-11* (Dpy) hermaphrodites. These *mes-4 dpy-11* (Dpy) hermaphrodites were mated to wild-type males. The Dpy progeny, the result of self-fertilization, were M-Z− (lacking both maternal and zygotic *mes-4*(+) product). The non-Dpy progeny, the result of cross-fertilization, were M-Z+. At least 24 of each category of progeny were scored for fertility (the production of embryos).

## Supporting Information

Figure S1 Specificity of newly developed monoclonal antibodies directed against H3K36me2 and H3K36me3. Specificity was analyzed by ELISA using histone H3 peptides containing different modifications. Microtiter plates coated with the indicated peptides (full sequences in Table 1 of [33]) were incubated with increasingly higher dilutions of each antibody (starting from 1∶100 dilution of a hybridoma culture supernatant). After incubation with peroxidase-conjugated secondary antibody and washing, the colorimetric signal of tetramethylbenzidine was detected by measuring the absorbance at 405 nm (Abs) using a plate reader.
(0.36 MB TIF)

Figure S2 Specificity of MES-4 ChIP reagents and distribution of MES-4 peaks on chromosomes. (A–C) One-cell embryos at pronuclear meeting were doubly stained with DAPI and SDI anti-MES-4 antibody (A, B) or triply stained with DAPI, anti-FLAG, and anti-MRG-1 (C). Arrows point to X chromosomes. (A) Wild-type embryo. (B) *mes-4(bn85)* embryo. The middle panel represents a longer exposure than that shown in A to demonstrate a complete lack of detectable MES-4. (C) *mes-4(bn73)* embryo carrying the MES-4::GFP::FLAG transgene. MRG-1 marks the autosomes. Scale bar: 5 µm. (D) For each chromosome the number of observed MES-4 peaks is given. The numbers of expected peaks were calculated assuming uniform distribution of all MES-4 peaks among chromosomes based on length. The number of observed peaks on X is highlighted with an asterisk.
(2.95 MB TIF)

Figure S3 High concordance between MES-4, MES-4::FLAG, Pol II, and H3K36me2/me3 ChIPs. (A) Genome browser view showing the reproducibility of MES-4 and MES-4::FLAG ChIPs and the similarity between MES-4 and MES-4::FLAG ChIP distributions. The z-scores of biological replicates and the average z-scores are shown across the same {similar, tilde operator } 180 kb region of ChrIV as shown in Figure 3. (B) Heatmap of Pearson correlation coefficients for ChIP biological replicates and ChIP performed against different targets. Correlations were calculated based on z-scores of all probes on the microarrays, after median smoothing over 250 bp. Green indicates positive correlation, red indicates anti-correlation, and white indicates no correlation. Numbers within the cells indicate rounded correlation values.
(2.82 MB TIF)

Figure S4 MES-4 bound genes largely overlap germline-expressed genes. (A) Venn diagram showing overlap of MES-4-bound genes with genes in the germline-expressed gene set. The overlap is enriched ∼3-fold over the overlap expected by chance (p-value<10^−300^). Notably, the average SAGE tag count in the germline SAGE library [34] of the 1906 germline-expressed genes not MES-4-bound is 1.6, whereas the average tag count of germline-expressed genes that are MES-4-bound is 14.6, indicating that germline-expressed genes not MES-4-bound are either very weakly expressed in the germline or possibly incorrectly classified as germline-expressed. (B) Venn diagram showing overlap of MES-4-bound genes with the embryo-expressed gene set. The overlap is ∼ 1/2 of that expected by chance (p-value ∼ 10^−13^). Of the 90 genes overlapping, 50 are actually either germline-expressed based on SAGE [34] or germline-enriched based on microarray analysis [21], strengthening the observed depletion of MES-4 from the embryo-expressed gene set. (C) Scatter plots of MES-4 vs Pol II (top row), H3K36me3 vs Pol II (middle row), and MES-4 vs H3K36me3 (bottom row) for all genes (gray) with ubiquitously-expressed (orange), germline-expressed (green), germline-specific (blue), and embryo-expressed (red) genes highlighted in the various columns. For each gene, a mean z-score was calculated by averaging the ChIP signal across all probes located between the transcript start and end site. Most striking are the low Pol II, high MES-4, high H3K36me3 pattern of many germline-specific genes, and the medium-high Pol II, low MES-4, low H3K36me3 pattern of many embryo-expressed genes.
(2.07 MB TIF)

Figure S5 MES-4 and H3K36 methyl marks show exonic enrichment. (A) Pol II, MES-4, H3K36me2, and H3K36me3 are elevated in exons compared to introns. This is especially apparent on genes with long introns. (B) Several ChIP targets, including MES-4 and H3K36me marks, increase across exon starts and decrease across exon ends. 2767 intron-exon-intron triplets with exons and introns of at least 300 bp were identified and binned by transcript level as in Figure 4. Profiles for MES-4 and other ChIPs are shown from 300 bp upstream to 200 bp downstream from exon start sites (St) and 200 bp upstream to 300 bp downstream from exon end sites (End). MES-4, Pol II, H3K36me2 and H3K36me3 levels are elevated in exons relative to introns in expressed genes. The latter was recently published [37]. (C) Analysis of separated IP and input signals. Profiles of log_2_ scores of IP and input over the same 2767 intron-exon-intron triplets as in (B) are shown. To better show exonic enrichment with respect to intronic signal, for each triplet the average log_2_ score of intronic probes was subtracted. MES-4, H3K36me3 and H3 IPs clearly show exonic enrichment; input shows significant but less exonic enrichment than IP. H3K9me3 IP also shows exonic enrichment; input shows even more exonic enrichment, leading to exonic depletion of log_2_ ratios of IP over Input for H3K9me3. NoAb Prot A beads and IgG also show exonic enrichment for both IP and input. Recent findings suggest that codon composition and/or higher GC content of exons bias them toward being occupied by nucleosomes [Schwartz S, Meshorer E, Ast G (2009) Chromatin organization marks exon-intron structure. Nat Struct Mol Biol 16: 990–995; Tilgner H, Nikolaou C, Althammer S, Sammeth M, Beato M, et al. (2009) Nucleosome positioning as a determinant of exon recognition. Nat Struct Mol Biol 16: 996–1001]. Increased nucleosome occupancy may in turn lead to higher levels of histone modifiers and histone modifications in exons. The fact that most of the ChIP signals we analyzed, including H3 and naked Protein G beads and even input chromatin, display some degree of exonic enrichment suggests that exonic DNA or more generally GC-rich DNA is preferentially solubilized during chromatin extraction and/or preferentially recovered during ChIP steps that depend on strength of base pairing [e.g. Quail MA, Kozarewa I, Smith F, Scally A, Stephens PJ, et al. (2008) A large genome center's improvements to the Illumina sequencing system. Nat Methods 5: 1005–1010].
(0.99 MB TIF)

Figure S6 Overlap between genes bound by MES-4 in embryos and genes mis-regulated in *mes-4* mutant germ lines. Based on transcript profiling of dissected adult germ lines from wild type and *mes-4* mutants, 352 X-linked genes and 270 autosomal genes are significantly mis-regulated (False Discovery Rate [FDR]<0.05) in *mes-4* mutants compared to wild type ([8] and our unpublished results). The overlap between mis-regulated genes and genes bound by MES-4 is small, but greater than expected by chance for two categories of genes: X-linked genes up-regulated in *mes-4* mutants, and autosomal genes down-regulated in *mes-4* mutants. There are numerous possible explanations for the relatively low overlap between MES-4-bound genes and genes mis-regulated in *mes-4* mutants. MES-4 may not affect expression of most of the genes on which it resides. MES-4 may directly influence a small number of genes, which in turn control other genes' expression. MES-4 may directly influence expression of most of its target genes, but 1) the full effect of loss of MES-4 requires analysis of PGCs instead of adult germ cells, 2) MES-4 only transiently associates with and regulates its target genes, and we failed to capture those transient associations, or 3) other factors mask the effect of loss of MES-4. Even well documented transcription regulators generally display a low overlap between genes bound and genes mis-regulated in mutants, for a variety of biological as well as technical reasons [e.g. 58], [59].
(0.60 MB TIF)

Table S1 Analysis of MES-4 binding sites. (A) Distribution of MES-4 peaks with respect to underlying genes. The number of MES-4 peaks that overlap with a gene body, within 1 kb 5′ or 3′ of the gene, or >1 kb away from a gene are given. (B) Number of MES-4 peaks whose maximum coordinate maps to an exon or intron. The middle coordinate of the probe with the maximum ChIP value within a MES-4 peak was mapped. P-values were calculated from a chi-square test between observed and expected distributions. Expected distributions were determined from all probes on the microarray that map to an exon or intron. (C) Gene Ontology terms that associate with MES-4-bound genes. Seven representative terms that significantly associate with MES-4-bound genes are shown.
(0.46 MB TIF)

Table S2 Lists of genes in different temporal-spatial classes. See Results for explanation of how the ubiquitous, germline-expressed, germline-specific, embryo-expressed, and soma-specific gene sets were derived from published microarray and SAGE data [21], [34]–[36]. Silent genes are from [37].
(0.16 MB XLS)

Table S3 List of genes with high MES-4 and H3K36me3 (z-scores>1) and low Pol II (z score<0).
(0.04 MB XLS)

Text S1 Supplemental methods.
(0.04 MB DOC)
