# Supplementary figures and images for: The Histone H3K36 Methyltransferase MES-4 Acts Epigenetically to Transmit the Memory of Germline Gene Expression to Progeny
Source: PLoS Genet. 2010 Sep 2;6(9):e1001091. doi: 10.1371/journal.pgen.1001091 (PMC2932692; doi:10.1371/journal.pgen.1001091)

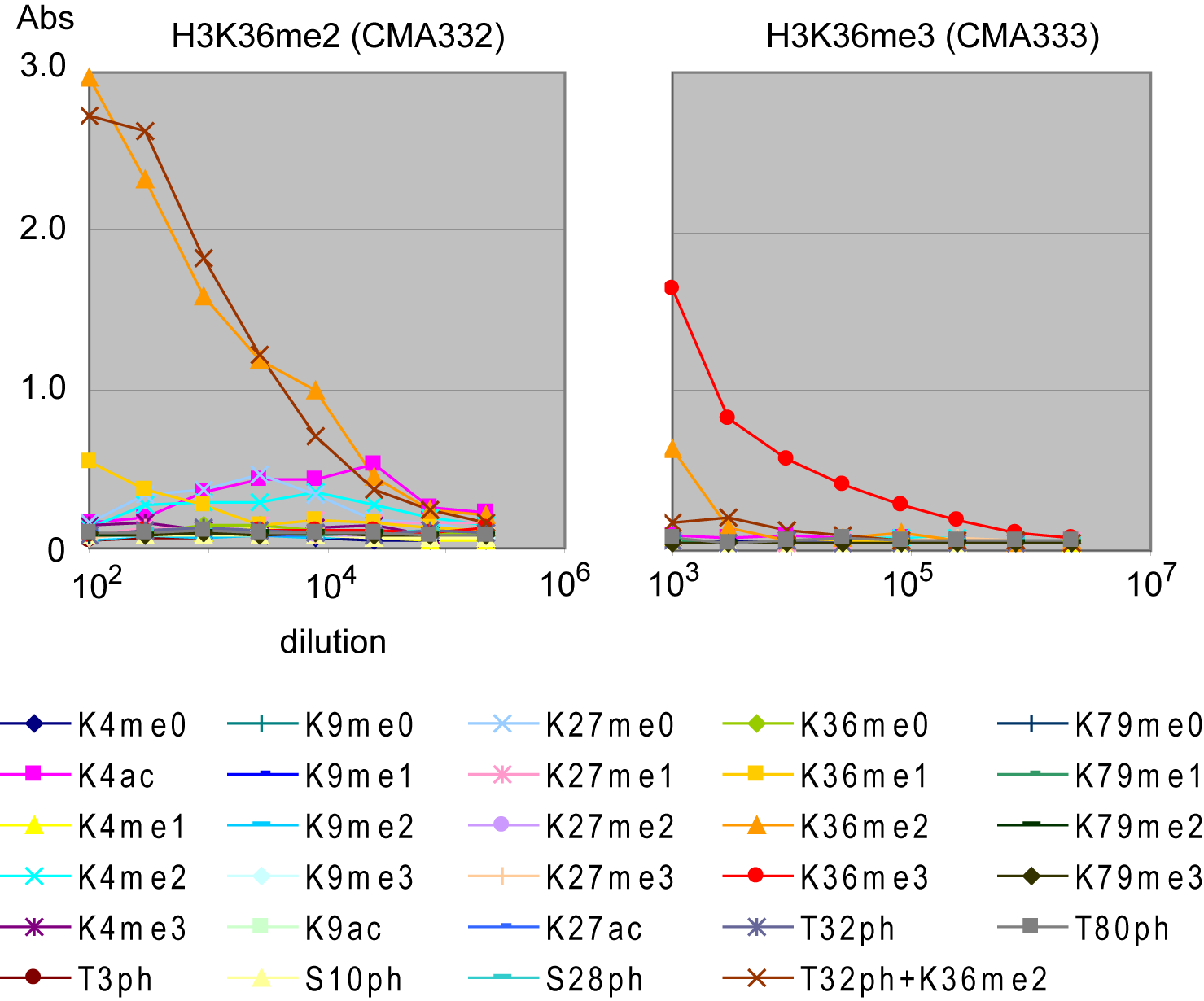

Supplement: Figure S1 — Specificity of newly developed monoclonal antibodies directed against H3K36me2 and H3K36me3. Specificity was analyzed by ELISA using histone H3 peptides containing different modifications. Microtiter plates coated with the indicated peptides (full sequences in Table 1 of [33]) were incubated with increasingly higher dilutions of each antibody (starting from 1∶100 dilution of a hybridoma culture supernatant). After incubation with peroxidase-conjugated secondary antibody and washing, the colorimetric signal of tetramethylbenzidine was detected by measuring the absorbance at 405 nm (Abs) using a plate reader. (0.36 MB TIF) [file pgen.1001091.s001.tif]

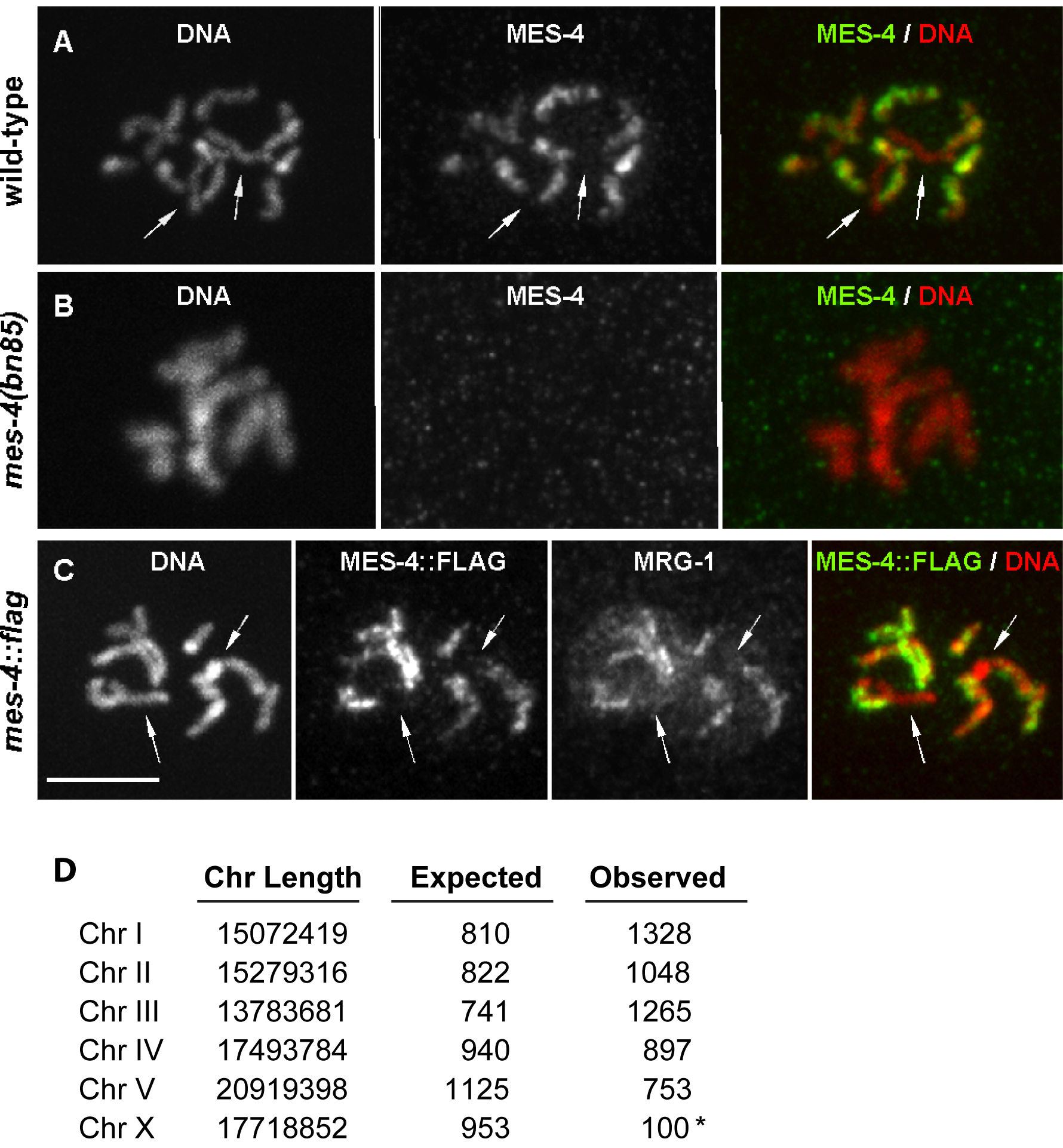

Supplement: Figure S2 — Specificity of MES-4 ChIP reagents and distribution of MES-4 peaks on chromosomes. (A–C) One-cell embryos at pronuclear meeting were doubly stained with DAPI and SDI anti-MES-4 antibody (A, B) or triply stained with DAPI, anti-FLAG, and anti-MRG-1 (C). Arrows point to X chromosomes. (A) Wild-type embryo. (B) mes-4(bn85) embryo. The middle panel represents a longer exposure than that shown in A to demonstrate a complete lack of detectable MES-4. (C) mes-4(bn73) embryo carrying the MES-4::GFP::FLAG transgene. MRG-1 marks the autosomes. Scale bar: 5 µm. (D) For each chromosome the number of observed MES-4 peaks is given. The numbers of expected peaks were calculated assuming uniform distribution of all MES-4 peaks among chromosomes based on length. The number of observed peaks on X is highlighted with an asterisk. (2.95 MB TIF) [file pgen.1001091.s002.tif]

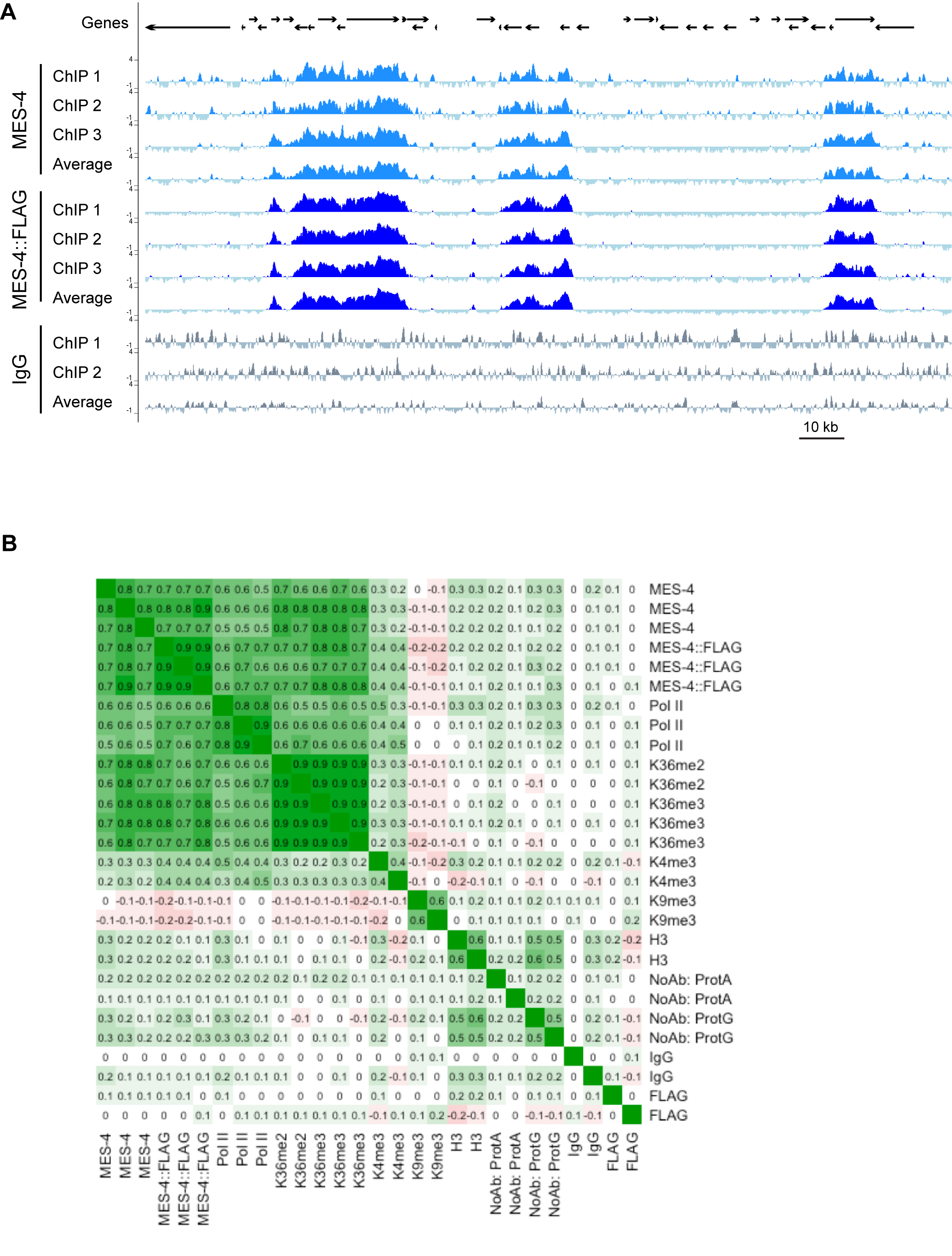

Supplement: Figure S3 — High concordance between MES-4, MES-4::FLAG, Pol II, and H3K36me2/me3 ChIPs. (A) Genome browser view showing the reproducibility of MES-4 and MES-4::FLAG ChIPs and the similarity between MES-4 and MES-4::FLAG ChIP distributions. The z-scores of biological replicates and the average z-scores are shown across the same {similar, tilde operator } 180 kb region of ChrIV as shown in Figure 3. (B) Heatmap of Pearson correlation coefficients for ChIP biological replicates and ChIP performed against different targets. Correlations were calculated based on z-scores of all probes on the microarrays, after median smoothing over 250 bp. Green indicates positive correlation, red indicates anti-correlation, and white indicates no correlation. Numbers within the cells indicate rounded correlation values. (2.82 MB TIF) [file pgen.1001091.s003.tif]

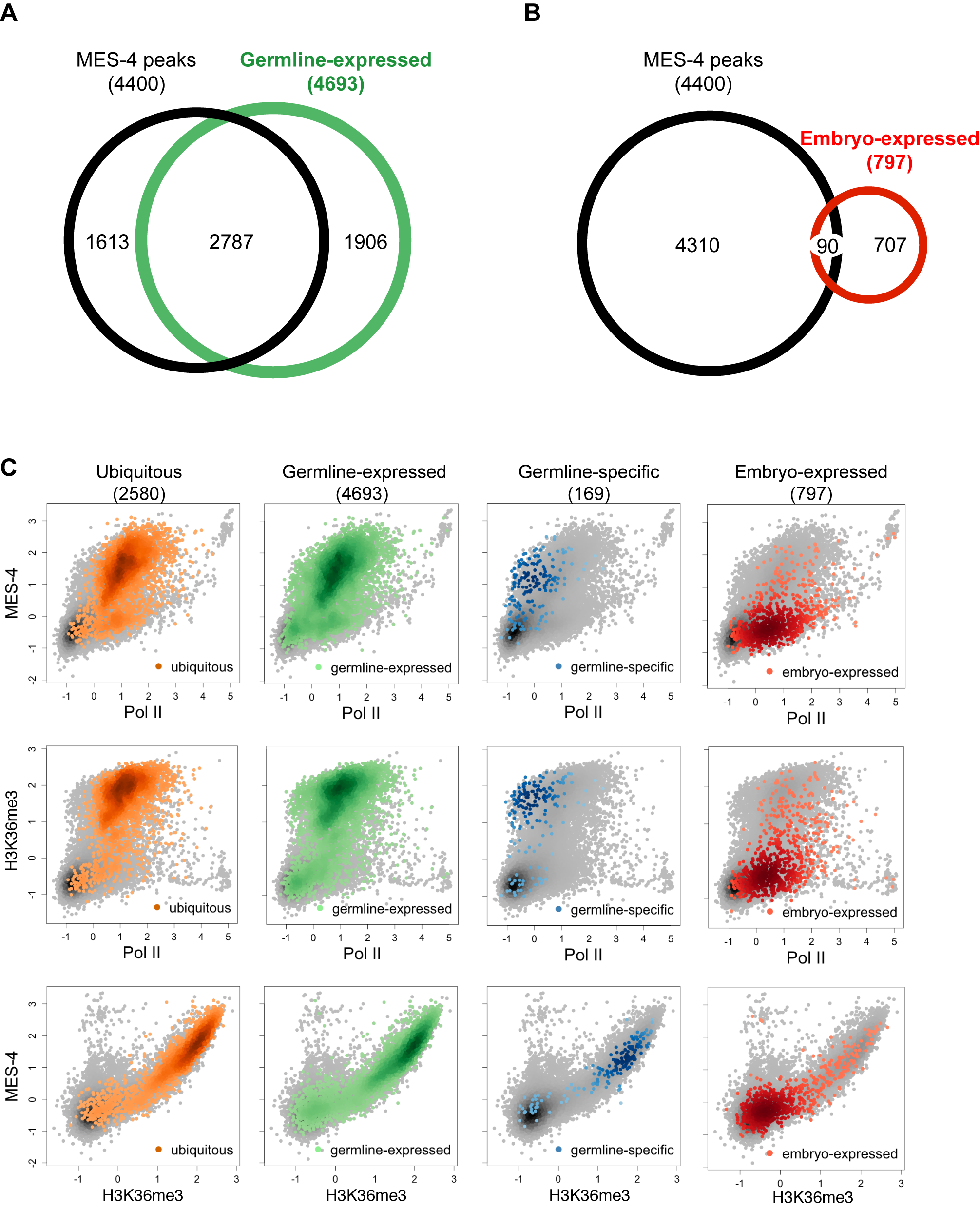

Supplement: Figure S4 — MES-4 bound genes largely overlap germline-expressed genes. (A) Venn diagram showing overlap of MES-4-bound genes with genes in the germline-expressed gene set. The overlap is enriched ∼3-fold over the overlap expected by chance (p-value<10−300). Notably, the average SAGE tag count in the germline SAGE library [34] of the 1906 germline-expressed genes not MES-4-bound is 1.6, whereas the average tag count of germline-expressed genes that are MES-4-bound is 14.6, indicating that germline-expressed genes not MES-4-bound are either very weakly expressed in the germline or possibly incorrectly classified as germline-expressed. (B) Venn diagram showing overlap of MES-4-bound genes with the embryo-expressed gene set. The overlap is ∼ 1/2 of that expected by chance (p-value ∼ 10−13). Of the 90 genes overlapping, 50 are actually either germline-expressed based on SAGE [34] or germline-enriched based on microarray analysis [21], strengthening the observed depletion of MES-4 from the embryo-expressed gene set. (C) Scatter plots of MES-4 vs Pol II (top row), H3K36me3 vs Pol II (middle row), and MES-4 vs H3K36me3 (bottom row) for all genes (gray) with ubiquitously-expressed (orange), germline-expressed (green), germline-specific (blue), and embryo-expressed (red) genes highlighted in the various columns. For each gene, a mean z-score was calculated by averaging the ChIP signal across all probes located between the transcript start and end site. Most striking are the low Pol II, high MES-4, high H3K36me3 pattern of many germline-specific genes, and the medium-high Pol II, low MES-4, low H3K36me3 pattern of many embryo-expressed genes. (2.07 MB TIF) [file pgen.1001091.s004.tif]

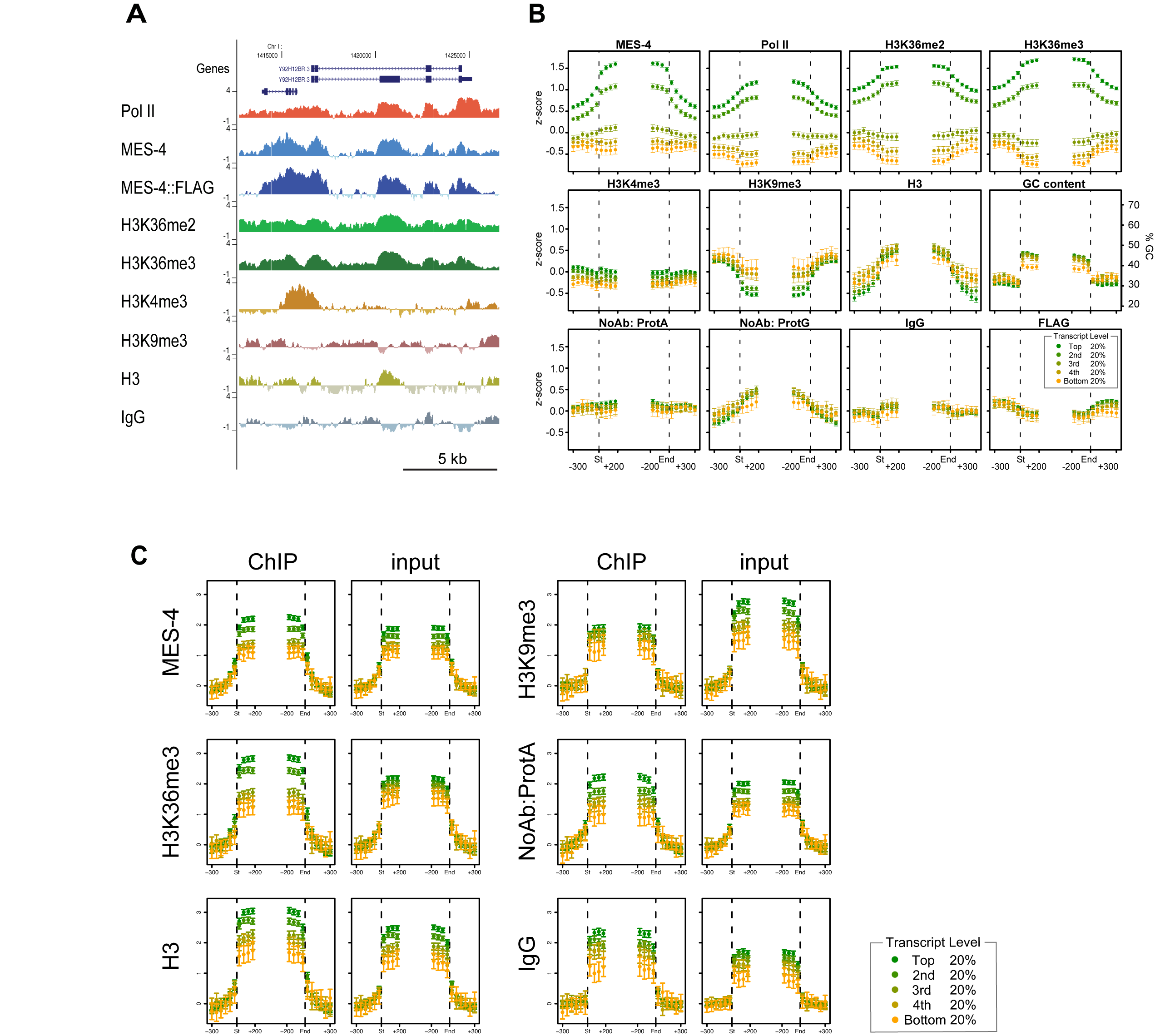

Supplement: Figure S5 — MES-4 and H3K36 methyl marks show exonic enrichment. (A) Pol II, MES-4, H3K36me2, and H3K36me3 are elevated in exons compared to introns. This is especially apparent on genes with long introns. (B) Several ChIP targets, including MES-4 and H3K36me marks, increase across exon starts and decrease across exon ends. 2767 intron-exon-intron triplets with exons and introns of at least 300 bp were identified and binned by transcript level as in Figure 4. Profiles for MES-4 and other ChIPs are shown from 300 bp upstream to 200 bp downstream from exon start sites (St) and 200 bp upstream to 300 bp downstream from exon end sites (End). MES-4, Pol II, H3K36me2 and H3K36me3 levels are elevated in exons relative to introns in expressed genes. The latter was recently published [37]. (C) Analysis of separated IP and input signals. Profiles of log2 scores of IP and input over the same 2767 intron-exon-intron triplets as in (B) are shown. To better show exonic enrichment with respect to intronic signal, for each triplet the average log2 score of intronic probes was subtracted. MES-4, H3K36me3 and H3 IPs clearly show exonic enrichment; input shows significant but less exonic enrichment than IP. H3K9me3 IP also shows exonic enrichment; input shows even more exonic enrichment, leading to exonic depletion of log2 ratios of IP over Input for H3K9me3. NoAb Prot A beads and IgG also show exonic enrichment for both IP and input. Recent findings suggest that codon composition and/or higher GC content of exons bias them toward being occupied by nucleosomes [Schwartz S, Meshorer E, Ast G (2009) Chromatin organization marks exon-intron structure. Nat Struct Mol Biol 16: 990–995; Tilgner H, Nikolaou C, Althammer S, Sammeth M, Beato M, et al. (2009) Nucleosome positioning as a determinant of exon recognition. Nat Struct Mol Biol 16: 996–1001]. Increased nucleosome occupancy may in turn lead to higher levels of histone modifiers and histone modifications in exons. The fact that most of the ChIP si [file pgen.1001091.s005.tif]

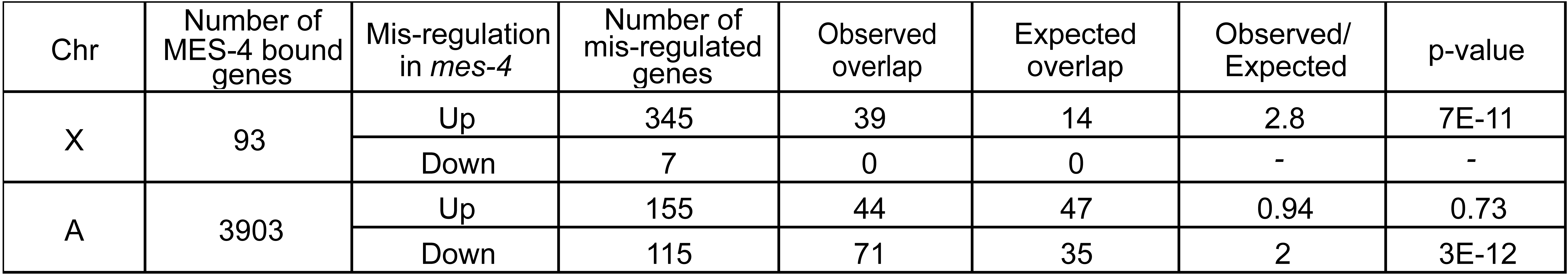

Supplement: Figure S6 — Overlap between genes bound by MES-4 in embryos and genes mis-regulated in mes-4 mutant germ lines. Based on transcript profiling of dissected adult germ lines from wild type and mes-4 mutants, 352 X-linked genes and 270 autosomal genes are significantly mis-regulated (False Discovery Rate [FDR]<0.05) in mes-4 mutants compared to wild type ([8] and our unpublished results). The overlap between mis-regulated genes and genes bound by MES-4 is small, but greater than expected by chance for two categories of genes: X-linked genes up-regulated in mes-4 mutants, and autosomal genes down-regulated in mes-4 mutants. There are numerous possible explanations for the relatively low overlap between MES-4-bound genes and genes mis-regulated in mes-4 mutants. MES-4 may not affect expression of most of the genes on which it resides. MES-4 may directly influence a small number of genes, which in turn control other genes' expression. MES-4 may directly influence expression of most of its target genes, but 1) the full effect of loss of MES-4 requires analysis of PGCs instead of adult germ cells, 2) MES-4 only transiently associates with and regulates its target genes, and we failed to capture those transient associations, or 3) other factors mask the effect of loss of MES-4. Even well documented transcription regulators generally display a low overlap between genes bound and genes mis-regulated in mutants, for a variety of biological as well as technical reasons [e.g. 58], [59]. (0.60 MB TIF) [file pgen.1001091.s006.tif]

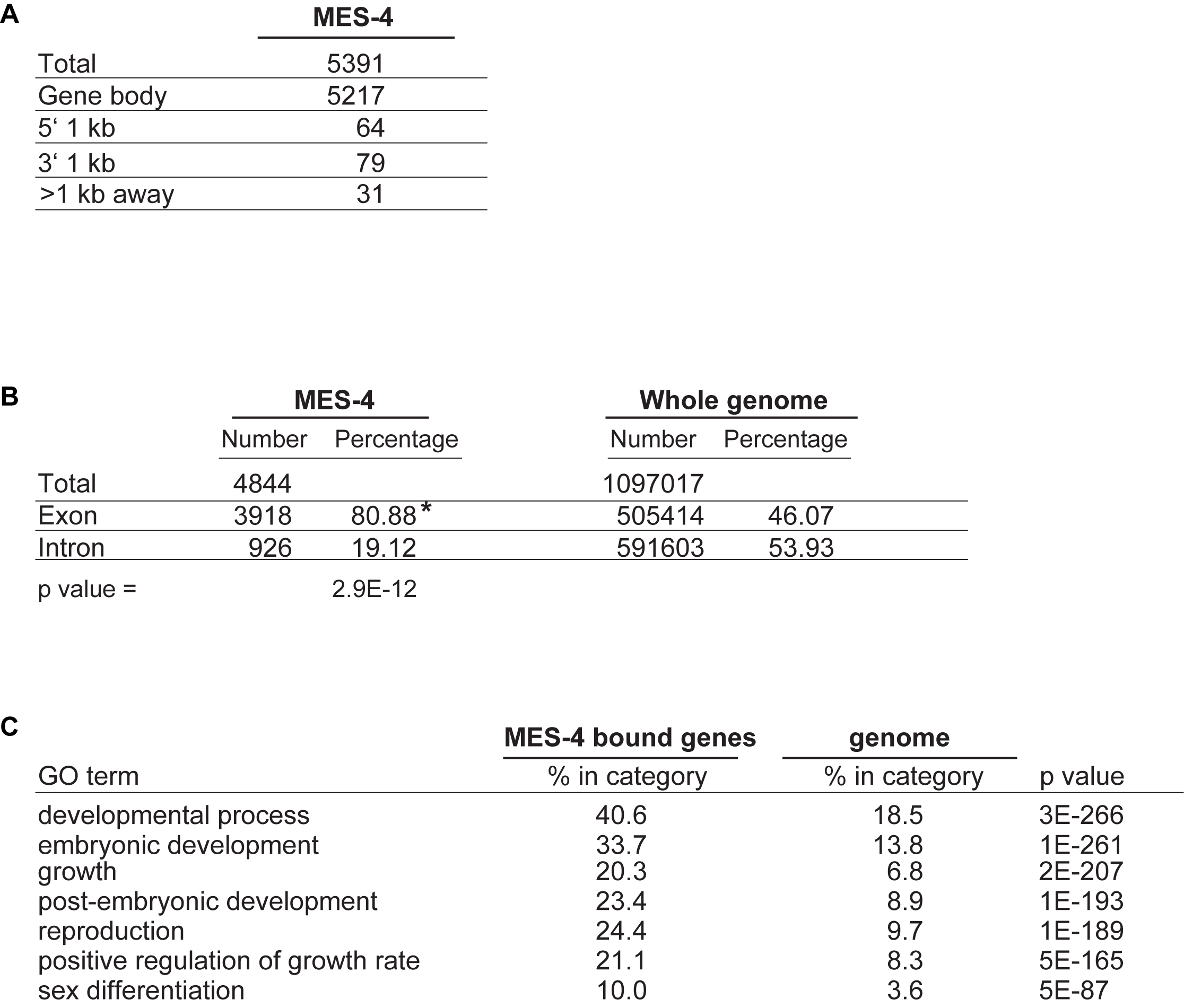

Supplement: Table S1 — Analysis of MES-4 binding sites. (A) Distribution of MES-4 peaks with respect to underlying genes. The number of MES-4 peaks that overlap with a gene body, within 1 kb 5′ or 3′ of the gene, or >1 kb away from a gene are given. (B) Number of MES-4 peaks whose maximum coordinate maps to an exon or intron. The middle coordinate of the probe with the maximum ChIP value within a MES-4 peak was mapped. P-values were calculated from a chi-square test between observed and expected distributions. Expected distributions were determined from all probes on the microarray that map to an exon or intron. (C) Gene Ontology terms that associate with MES-4-bound genes. Seven representative terms that significantly associate with MES-4-bound genes are shown. (0.46 MB TIF) [file pgen.1001091.s007.tif]
