# Supplementary material for: The Histone H3K36 Methyltransferase MES-4 Acts Epigenetically to Transmit the Memory of Germline Gene Expression to Progeny
Source: PLoS Genet. 2010 Sep 2;6(9):e1001091. doi: 10.1371/journal.pgen.1001091 (PMC2932692; doi:10.1371/journal.pgen.1001091)
Supplement: Text S1 — Supplemental methods. (0.04 MB DOC) [file pgen.1001091.s010.doc]

**Supplemental methods.**

**Generation of transgenic animals expressing MES-4::GFP::3xFLAG**

To generate an epitope-tagged version of MES-4, we created a transgene containing the *mes-3* promoter (2903 bp) followed by *mes-4* genomic coding sequence (4065 bp), GFP coding sequence with 3 artificial introns (867 bp), 3xFLAG coding sequence (66 bp), and *mes-4* 3′ region (2390 bp) in pUC19 vector (Invitrogen). Transgenic strains were generated by microparticle bombardment of *unc-119* worms (Praitis et al., 2001) with the plasmid described above together with pJN254, a vector containing *unc-119*(+) genomic DNA (from J. Nance). The integrated transgenic line *bnIs15*, identified by stable passage of *unc-119(+)* to progeny, displayed GFP expression that mimicked the endogenous expression pattern of MES-4. Transgenic *bnIs15* males were mated to *mes-4(bn73)dpy-11(e224)/DnT1* hermaphrodites to obtain *mes-4(bn73) dpy-11(e224)/++;* *mes-4::gfp::3xflag/+* progeny. From those heterozygotes, Dpy progeny that yielded stably-maintained GFP expression in 100% of their offspring were obtained. Removal of the *dpy-11* marker was accomplished by crossing hermaphrodites with *mes-4(bn73)/DnT1[qls50]* males. The genotype of the resulting *mes-4(bn73); bnIs15[mes-4::gfp::3xflag]* strain was confirmed by sequencing the endogenous and transgenic *mes-4* genes.

**Chromatin immunoprecipitation (ChIP) of MES-4::GFP::3xFLAG**

MES-4::GFP::3xFLAG was ChIPed using 6 mg embryo protein and 5 μg antibody. For each ChIP reaction, antibody was incubated overnight at 4°C with Protein G- or Protein A-coupled Dynabeads (Invitrogen), respectively, in 100 μl of 5 mg/ml IgG-free BSA (Sigma) in PBS. Antibody-coated beads were washed twice with 5 mg/ml IgG-free BSA, added to 1 ml FA buffer containing the chromatin, nutated for 2 hr at 4°C, concentrated using a Dynal Magnetic Particle Concentrator (MPC) (Invitrogen) and washed at room temperature for 5 min in 1 ml of each of the following buffers: FA buffer, 3 washes; FA buffer with 500 mM NaCl, 2 washes; and FA buffer with 1M NaCl, 1 wash. Beads were transferred to a new tube, washed once for 5 min at room temperature in 1 ml RIPA buffer (10 mM Tris-HCl pH 8.0, 1 mM EDTA, 250 mM LiCl, 0.5% sodium deoxycholate, 0.5% NP40) and twice for 5 min at room temperature in 1 ml TE. To elute complexes, beads were incubated twice at 65°C in 150 μl of elution buffer (1% SDS in TE with 250 mM NaCl) for 15 min. Input samples were brought to a volume of 300 μl. Both input and IP samples were incubated with 20 μg proteinase K for 1 hr at 55°C. Crosslinks were reversed overnight at 65°C. DNA was purified using a Zymo DNA purification column.

**Exon-intron analysis**

Intron-exon-intron profile plots were generated similarly to the gene body profile plots. Intron-exon-intron triplets with exons and introns ≥ 300 bp were identified in WormBase 170 (2676 triplets from 1763 genes). Exons and introns were aligned at their intron-exon and exon-intron junctions. The genomic regions 300 bp upstream to 200 bp downstream from the intron-exon junction and 200 bp upstream to 300 bp downstream from the exon-intron junction were divided into 10 50-bp bins each. Probes in those genomic regions were assigned to the nearest bin. To display the IP channel and input channel separately across intron-exon junctions, the average z-score of the probes within the 2 introns was subtracted from the z-score of all probes within the triplet.

**GO term analysis**

MES-4-bound genes (4400 determined from MES-4 ChIP) were analyzed with the GO Term Finder (<http://go.princeton.edu/>). 1543 genes were ambiguous or unidentified in the GO Term database, 2857 were used. Seven representative terms were selected from the top 20 GO terms. These are shown in Table S1C, with the percentage of genes within the MES-4-bound genes and within all genes, and the associated multiple hypotheses corrected p-value.

**Supplemental reference**

Praitis V, Casey E, Collar D, Austin J (2001) Creation of low-copy integrated transgenic lines in *Caenorhabditis elegans*. Genetics 157: 1217-1226.
